# Evidence of pyroptosis and ferroptosis extensively involved in autoimmune diseases at the single-cell transcriptome level

**DOI:** 10.1186/s12967-022-03566-6

**Published:** 2022-08-12

**Authors:** Danfeng Zhang, Yadan Li, Chunyan Du, Lina Sang, Liu Liu, Yingmei Li, Fang Wang, Wenjuan Fan, Ping Tang, Sidong Zhang, Dandan Chen, Yanmei Wang, Xiaoyi Wang, Xinsheng Xie, Zhongxing Jiang, Yongping Song, Rongqun Guo

**Affiliations:** 1 Department of Hematology, The First Affiliated Hospital of Zhengzhou University, Zhengzhou, Henan China; 2 Academy of Medical Science, Henan Medical College of Zhengzhou University, Zhengzhou, Henan China; 3 Laboratory Animal Center, School of Medical Sciences, Zhengzhou University, Zhengzhou, Henan China; 4 Department of Pediatric Hematology and Oncology, The First Affiliated Hospital of Zhengzhou University, Zhengzhou, China; 5 Department of Hematology, Zhengzhou People’s Hospital, Zhengzhou, Henan China

## Abstract

**Background:**

Approximately 8–9% of the world’s population is affected by autoimmune diseases, and yet the mechanism of autoimmunity trigger is largely understudied. Two unique cell death modalities, ferroptosis and pyroptosis, provide a new perspective on the mechanisms leading to autoimmune diseases, and development of new treatment strategies.

**Methods:**

Using scRNA-seq datasets, the aberrant trend of ferroptosis and pyroptosis-related genes were analyzed in several representative autoimmune diseases (psoriasis, atopic dermatitis, vitiligo, multiple sclerosis, systemic sclerosis-associated interstitial lung disease, Crohn’s disease, and experimental autoimmune orchitis). Cell line models were also assessed using bulk RNA-seq and qPCR.

**Results:**

A substantial difference was observed between normal and autoimmune disease samples involving ferroptosis and pyroptosis. In the present study, ferroptosis and pyroptosis showed an imbalance in different keratinocyte lineages of psoriatic skinin addition to a unique pyroptosis-sensitive keratinocyte subset in atopic dermatitis (AD) skin. The results also revealed that pyroptosis and ferroptosis are involved in epidermal melanocyte destruction in vitiligo. Aberrant ferroptosis has been detected in multiple sclerosis, systemic sclerosis-associated interstitial lung disease, Crohn’s disease, and autoimmune orchitis. Cell line models adopted in the study also identified pro-inflammatory factors that can drive changes in ferroptosis and pyroptosis.

**Conclusion:**

These results provide a unique perspective on the involvement of ferroptosis and pyroptosis in the pathological process of autoimmune diseases at the scRNA-seq level. IFN-γ is a critical inducer of pyroptosis sensitivity, and has been identified in two cell line models.

**Supplementary Information:**

The online version contains supplementary material available at 10.1186/s12967-022-03566-6.

## Introduction

Cell death plays a critical role in embryonic development, cell fate determination, and maintenance of immune homeostasis and is categorized into necroptosis, pyroptosis, apoptosis, ferroptosis, and necrosis [1]. However, the distinction between these cell death modalities remains to be further investigated. Necroptosis may be the pathogenesis of inflammatory bowel disease (IBD), Crohn’s disease, skin inflammation, severe cutaneous adverse drug reactions, alcohol/ non-alcoholic steatohepatitis, and drug-induced liver injury. It may be triggered by TLR4/TLR3 stimulation, DNA-dependent activator of IFN regulatory factors (DAI), TNF-TNFR1 signaling, FASL-FAS signaling, TRAIL-TRAILR signaling, and IFN-α/β-IFNAR1 signaling, and intertwined with apoptosis [2]. Apoptosis, the most studied type of “programmed or regulated cell death” (PCD), ranges from inflammation and cancer to autoimmune diseases mediated by FASL-FAS signaling [3]. Unlike apoptosis, necrosis involves the release of intracellular contents and elicits acute exudative inflammation in surrounding tissues [4]. Ferroptosis and pyroptosis have been recently identified as forms of programmed cell death amd have become novel potential therapeutic targets for cancer therapy [5, 6].

Ferroptosis, an iron-dependent cell death modality, leads to toxic accumulation of reactive oxygen species. Dysregulation of iron metabolism and phospholipid peroxidation can lead to ferroptosis. Owing to its unique mechanism, ferroptosis might be involved in cell fate decisions, inflammatory progress, and several pathologies, such as cardiomyopathy [7] and acute kidney injury [8]. A growing body of research supports the notion that ferroptosis is involved in the response to immunotherapy [6], such as the different sensitivities of immune cell types and the increasing ferroptosis sensitivity of target cells mediated by IFN-γ [9]. Therefore, regulating the sensitivity to ferroptosis in cancer cells can reshape the tumor microenvironment, which can be targeted in antitumor therapy [10]. However, it remains unclear whether ferroptosis is related to dysregulated immune responses. Ferroptosis has been previously reported to trigger proinflammatory state in the development of necroinflammatory diseases [11]. Dysregulated immune systems also lead to autoimmune diseases, such as systemic lupus erythematosus (SLE) and multiple sclerosis (MS). The relationship between inflammatory progress and ferroptosis prompted us to investigate whether ferroptosis is involved in the development of autoimmune diseases [12]. In the S100-induced autoimmune hepatitis (AIH) mouse model, ferroptosis caused hepatitis by downregulating glutathione peroxidase 4 (GPX4), and was inhibited by the Ferrostatin-1 mediated Nrf2/HO-1 signaling pathway [13]. Ferroptosis also exists in concanavalin A-induced AIH [14]. Recent evidence has shown that neutrophil ferroptosis is a key driver of neutropenia in SLE, whichis mediated by autoantibodies and interferon-α (IFN-α). As a result, GPX4 expression is downregulated and elevation of lipid-reactive oxygen species is induced [15]. However, the pathological role of ferroptosis in autoimmune diseases has rarely been explored.

Pyroptosis is a newly discovered modality of programmed cell death that is mediated by pyroptotic caspases. The modality features rapid plasma-membrane rupture and initiates the release of proinflammatory intracellular contents [16]. While pathogen-associated molecular patterns (PAMPs) and LPS are recognized by corresponding inflammasomes and caspases, such as caspase-1 (CASP1), caspase-3 (CASP3), caspase-11 (Casp11) and counterpart caspase-4 (CASP4), and caspase-5 (CASP5) in human, respectively to induce the activation of pyroptosis pathways, increasing evidence suggests that pyroptosis is deeply involved in infectious diseases [17], hematologic disorders [18], and tumorigenesis [19]. In addition, PAMPs and damage-associated molecular pattern (DAMPs)-induced activated inflammasomes are involved in the initiation of chronic inflammation and autoimmune diseases [17], which also initiate pyroptosis [20]. Many factors, including pore-forming toxin, protease, ubiquitin ligase, Rho-inactivating toxin, dsDNA, and flagellin, can activate caspase-1, and LPS activates caspase-4/5/11, which subsequently cleaves GSDMD, leading to the formation of lethal pores and the release of proinflammatory factors [21]. A landmark study showed that interferon-γ (IFN-γ) upregulated GSDMB expression in target cells and granzyme A (GZMA) secreted by cytotoxic lymphocytes (CTLs) cleaved GSDMB to induce pyroptosis [22]. The CTLs participate in autoimmune and degenerative central nervous system (CNS) diseases, such as MS and amyotrophic lateral sclerosis (ALS). Hyperactive CTLs causes cell death in various CNS cell types to some extent via cytotoxic granule-induced membrane lysis, Fas ligand, TNF, and related molecules [23]. Interestingly, caspase-3 or CTL-secreted granzyme B (GZMB) cleaves gasdermin E (GSDME) at the same site, which activates the pyroptosis of target cells [24]. Previous studies have shown that GSDMB-induced excessive pyroptosis might lead to autoimmune disorders, such as asthma and inflammatory bowel disease (IBD) [25]. Based on this, pyroptosis is considered to have implications in many autoimmune disorders [26], however, this aspect remains understudied.

In the present work, of the goal was to investigate the mechanisms in which ferroptosis and pyroptosis are involved in several autoimmune diseases (psoriasis, atopic dermatitis [AD], vitiligo, multiple sclerosis [MS], systemic sclerosis-associated interstitial lung disease [SSc-ILD], Crohn’s disease [CD], and experimental autoimmune orchitis [EAO]) at the single cell RNAseq (scRNA-seq) level. scRNA-seq is a powerful tool for characterizing physiological and pathological processes at the single-cell level [27–29]. Notably, among the results, we confirmed the sensitivity of ferroptosis and pyroptosis in different target cell types of different autoimmune diseases, such as cutaneous keratinocytes of patients with AD. Imbalances in ferroptosis and pyroptosis during the development of psoriasis were also identified. In addition, vitiligo may be driven by pyroptosis and ferroptosis of epidermal melanocytes. Ferroptosis is involved in the pathologies of MS, SSc-ILD, CD, and EAO. Interestingly, results showed that the proinflammatory factor IFN-γ increased the sensitivity of pyroptosis in the B16 cell line model and THP-1 differentiation model, but had less effect on ferroptosis, which was dependent on concentration and exposure time. In summary, our results provide a link between autoimmune diseases and ferroptosis and pyroptosis.

## Methods

### scRNA-seq datasets

The scRNA-seq datasets of 5 healthy donor skin samples and 8 vitiligo-affected skin samples were acquired from the Genome Sequence Archive (GSA) with accession number PRJCA006797 (https://ngdc.cncb.ac.cn/bioproject/browse/PRJCA006797) (Table 1). The scRNA-seq datasets of lesional/non-lesional skin biopsies of AD patients were obtained from GEO database (Accession NO. GSE147424). The datasets of skin biopsy tissues from 13 psoriasis patients and 5 healthy volunteers were acquired from GSE151177. Single nuclei RNA-sequencing (snRNA-seq) datasets of brain samples of control and MS patients were obtained from GSE118257. And the droplet-based scRNA-seq datasets of human healthy and SSc-ILD lungs were got from GSE128169. The scRNA-seq datasets of terminal ileum of childhood onset Crohn’s disease and matched healthy controls were downloaded from https://cellgeni.cog.sanger.ac.uk/gutcellatlas/pediatric_RAWCOUNTS_cellxgene.h5ad. And the scRNA-seq datasets of unilateral decapsulated testes from mouse experimental and control group were obtained from GSM5563668 and GSM5563669.

**Table 1:** Information about the scRNA-seq datasets used in our study

Organs/Tissues	ID	Database
Vitiligo-affected skin	PRJCA006797	Genome Sequence Archive (GSA)
AD-affected skin	GSE147424	GEO
Psoriasis-affected skin	GSE151177	GEO
MS-affected brain	GSE118257	GEO
SSc-ILD lung	GSE128169	GEO
CD-affected terminal ileum	https://cellgeni.cog.sanger.ac.uk/gutcellatlas/pediatric_RAWCOUNTS_cellxgene.h5ad	https://www.gutcellatlas.org/
Unilateral decapsulated testes	GSM5563668 and GSM5563669	GEO

### Quality control

Cells from psoriasis patients and healthy volunteers were filtered with a gene expression number per cell between 200 and 10,000, and the mitochondrial percentage per cell was below 50. Cells from the vitiligo-affected skin samples and healthy skin samples were filtered with a gene expression number per cell between 200 and 10,000, and the mitochondrial percentage per cell was below 25. And cells from lesional/non-lesional skin biopsies of AD patients were filtered with a gene expression number per cell between 200 and 10,000. Quality control of cells from brain samples was applied to filter out low-quality cells with < 200 or > 10,000 expressed genes. Cells from lung samples were filtered with a gene expression number per cell between 200 and 10,000, and the mitochondrial percentage per cell was below 25. The processed dataset of terminal ileum was converted into h5Seurat data, and used without any additional filtration. And quality control of cells from decapsulated testes was applied to filter out low-quality cells with either (1) < 200 or > 10,000 expressed genes or (2) < 25 mitochondrial percentage.

### Data processing

Datasets of vitiligo skin biopsies, psoriasis skin biopsies, AD skin biopsies, MS brain cells, SSc-ILD lung biopsies, decapsulated testes, and its counterparts, were processed in Seurat V4, integrated by “merge” function, and normalized by “NormalizeData” function. Then “FindVariableFeatures”, “ScaleData”, “RunPCA”, “FindNeighbors”, and “SCTransform” were used to process the datasets. Next, “RunHarmony (object, group.by.vars = ”orig.ident”)” and “RunUMAP(object, reduction = ”harmony” were used, while clusters were calculated using the “FindClusters” function with a resolution of 0.5. The Seurat file of terminal ileum was used without any additional data processing.

### Separation and identification of cell types

**Fig. 1:**
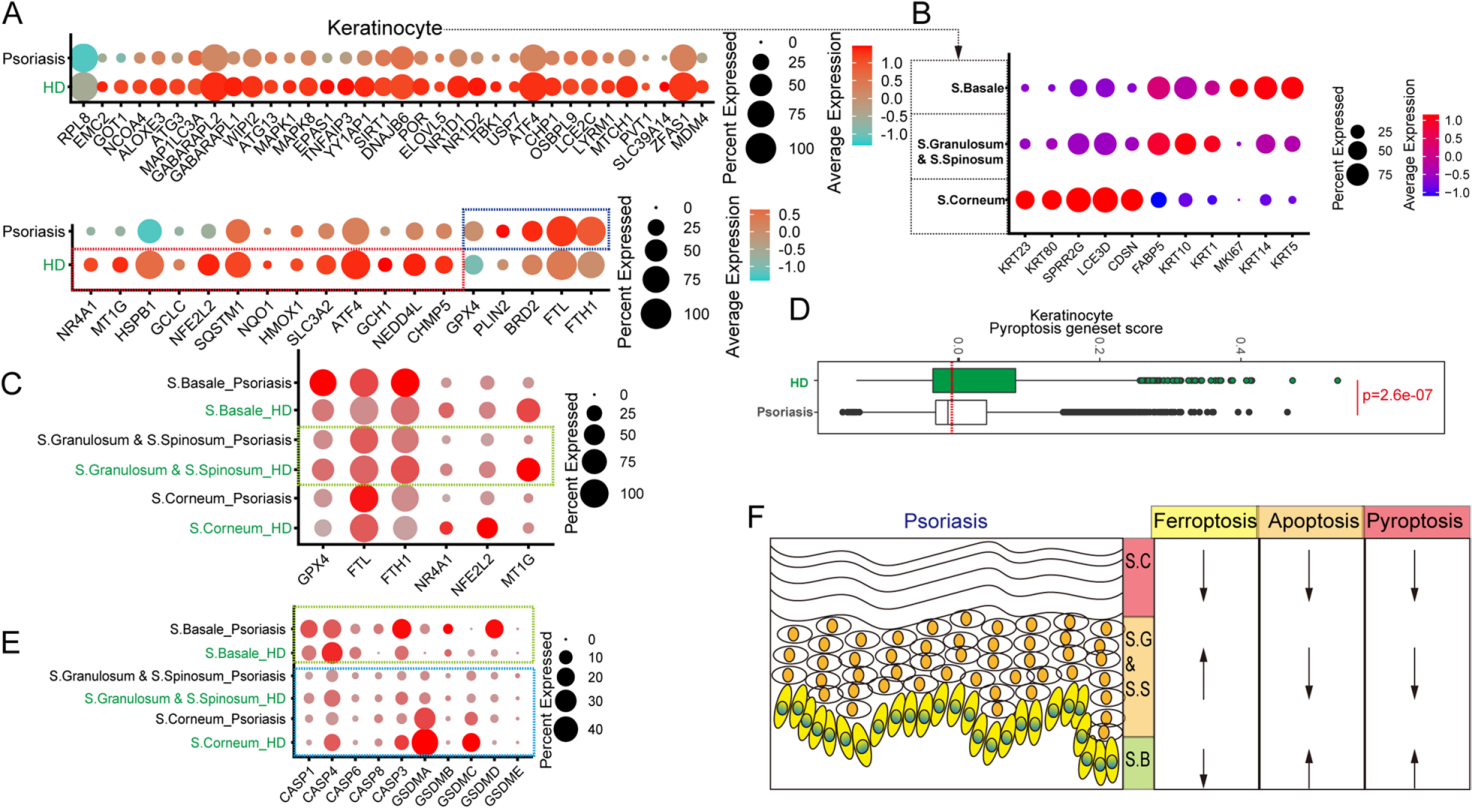
The expression patterns of ferroptosis and pyroptosis-related genes in different keratinocyte subtypes. **(A)** Dot plot shows the expression levels of pro-ferroptosis and ferroptosis-resistant genes in total keratinocytes derived from psoriasis skin and healthy skin. **(B)** Dot plot shows the expression levels of feature genes in different keratinocyte subsets. **(C)** The expression levels of GPX4, FTL, FTH1, NR4A1, NFE2L2, and MT1G in different subsets. **(D)** Quantification of pyroptosis geneset score of keratinocytes in psoriasis skin and healthy skin. **(E)** Dot plot shows the expression levels of CASP1, CASP4, CASP6, CASP8, CASP3, GSDMA, GSDMB, GSDMC, GSDMD, and GSDME in different keratinocyte subsets derived from psoriasis skin and healthy skin. **(F)** Schematic depiction of the relationship of psoriasis pathological process with ferroptosis/apoptosis/pyroptosis

Cluster of Melanocytes was characterized by the high expression of *DCT*, *TYRP1*, *PMEL*, and *MLANA* in vitiligo skin and its counterparts-derived cells. Ketatinocytes were identified by the high expression of *KRT14*, *KRT1*, *KRT10*, and *KRT15*. Fibroblasts were identified by feature genes including *COL1A1*, *DCN*, *SFRP2*, and *TWIST2*, while other cell types were identified by below genesets, (1) smooth muscles: *TAGLN*, *ACTA2*, and *NR2F2*; (2) ECs: *AQP1*, *CLEC14A*, *PECAM1*, and *ECSCR.1*; (3) Langerhans: *PCGBP*, *CD207*, *CD1A*, and *S100B*; (4) T cells: *PTPRC*, *CD3D*, *CD3E*, *TRBC2*, *TRAC*, and *TRGC2*; (5) myeloid cells: *PTPRC*, *CD14*, *FCGR3A*, and *CD1C*. Ketatinocytes of psoriasis skin biopsies and its counterparts were further identified according to S.Basale, S.Granulosum&S.Spinosum, and S.Corneum based on the expression of signature genes presenting on Fig. 1B. Clusters of AD skin biopsies and its counterpart-derived cells were characterized by feature genes described in Additional file 2: Fig. S2B, then renamed and merged into several major clusters by the “RenameIdents” function.

Clusters of OPCs were characterized by the high expression of *SOX6*, *OLIG2*, *PDGFRA*, and *BCAN* in brain cells. Neurons were identified by the high expression of *SNAP25* and *GABRB2*. Oligodendrocytes were identified by the feature genes *PLP1* and *CNP*. And astrocytes were characterized by the high expression of *APOE*, *GJA1*, and *AQP4*, while other cell types were identified by below genesets, (1) immune cells: *PTPRC*, *CD14*, and *IGHM*; (2) EC/VSM: *CLEC14A* and *PECAM1*.

Macrophages were identified by the high expression of *CD14*, *FCGR3A*, *ITGAX*, *SPP1*, *APOE*, *FABP4*, and *IL1B* in lung-derived cells. Club/Goblet/Basal cells were characterized by the high expression of *CAPS* and *TPPP3*. Alveolar Type 1/2 cells were identified by the high expression of *SCGB3A1*, *SLPI*, *SFTPA1*, and *SFTPC*. Other cell types were identified by below genesets, (1) ECs: *CD34*, *FCN3*, *CLDN5*, *ACKR1*, and *VWF*; (2) Fibroblasts: *DCN* and *LUM*; (3) Smooth Muscles/Pericytes: *TAGLN* and *ACTA2*; (4) Mast cells: *TPSAB1* and *TPSB2*.

Epithelial cells featured the high expression of *EPCAM* and *FABP1* in terminal ileum-derived cells. Fibroblasts were identified by the high expression of *COL1A1*, *COL1A2*, and *DCN*. And myeloid cells were identified by these feature genes of *PTPRC*, *CD14*, *FCGR3A*, *ITGAM*, and *CSF1R*. Clusters of decapsulated testes were characterized by those feature genes showed in Additional file 7: Fig. S7B.

### Calculating module scores

The AddModuleScore function from Seurat v4.0 was used to score target cells according to the expression of signature gene lists. We grouped the expression values of human ferroptosis driver gene set (Additional file 8: Table S1) [30], constructed a signature that we named the “ferroptosis driver” module, and scored target cell types using the “AddModule Score” function. And we grouped the expression values of human ferroptosis suppressor gene set (Additional file 9: Table S2), constructed a signature that we named the “ferroptosis suppressor” module. What’s more, the mouse gene lists were by Additional file 10: Table S3 and Additional file 11: Table S4. And finally, we grouped the expression values of these pyroptosis-related genes (mouse: *Casp1*, *Casp11*, *Casp6*, *Casp8*, *Gsdma1*, *Gsdma2*, *Gsdma3*, *Gsdmc*, *Gsdmd*, and *Gsdme*; human: *CASP1*, *CASP4*, *CASP6*, *CASP8*, *GSDMA*, *GSDMB*, *GSDMC*, *GSDMD*, and *GSDME*), constructed a signature that was named the “pyroptosis” module. These geneset scores were showed as violin plots and box-plots.

### Quantitative PCR analysis

B16 cell line was treated with mouse IFN-γ (5 ng/mL or 10 ng/mL) (C746, Novoprotein Scientific) and collected at different time points (12 h, 24 h, and 48 h). Total RNA was isolated from Trizol (15596026, Invitrogen) treated cells. cDNA was synthesized using HiScript II Q RT SuperMix for qPCR (+ gDNA wiper) kit (R223-0, Vazyme). qPCR was performed on cDNA using Hieff qPCR SYBR Green Master Mix (Low Rox Plus) (11202ES08, YEASEN). Gene expression was quantified using the following primers: mouse *Gapdh* forward: CAGTGGCAAAGTGGAGATTGTTG; mouse *Gapdh* reverse: TCGCTCCTGGAAGATGGTGAT; mouse *Gsdmd* forward: CACCATGGCCTCAATGTGCT; mouse *Gsdmd* reverse: GCAAGCCTTCACCTCAGCAT; mouse *Gsdme* forward: TGCAACTTCTAAGTCTGGTGACC; mouse *Gsdme* reverse: CTCCACAACCACTGGACTGAG; mouse *Casp1* forward: GCCGTGGAGAGAAACAAGGAGTG; mouse *Casp1* reverse: TCAATGAAAAGTGAGCCCCTGACAG; mouse *Casp8* forward: TCCTGTGCTTGGACTACATCC; mouse *Casp8* reverse: TTCCCGCAGCCTCAGAAATAG; mouse *Gpx4* forward: ATAAGAACGGCTGCGTGGTGAAG; mouse *Gpx4* reverse: TAGAGATAGCACGGCAGGTCCTTC; mouse *Slc7a11* forward: TGGCGGTGACCTTCTCTGA; mouse *Slc7a11* reverse: ACAAAGATCGGGACTGCTAATGA; mouse *Slc3a2* forward: TGCTCAGGCTGACATTGTAGC; mouse *Slc3a2* reverse: TCAGCCAAGTACAAGGGTGC. Fold changes in mRNA expression were calculated by the ΔΔCt method using Gapdh as an endogenous control. Results are expressed as fold change by normalizing to the controls. And the expression of Gsdmc, Casp6, Gsdma, Gsdma2, Gsdma3, Casp11, and Ascl4 was too low and didn’t present.

### THP-1 differentiation and bulk RNA-seq analysis

The THP-1 cells were cultured in RPMI1640 medium supplemented with 10% FBS and 1 × P/S. And THP-1 cells were seeded into 25cm^2^ flasks at the density of 5 × 10^5^ cells/flask and were cultured with 10 ng/mL PMA (abs9107, absin) for 24 h. Then these cells were cultured with medium without stimulatory factor, or supplemented with IFN-γ (50 ng/mL)( abs04123, absin) and LPS (15 ng/mL)( L8880, Solarbio), or only IFN-γ(50 ng/mL) for further 5 days. RNA were extracted from 1 × 10^6^ THP-1 cells or THP-1-derived cells of each sample, and sequenced using RNA-seq platform at Guangzhou Huayin Health Medical Group. Sequencing was run on an Illumina HiSeqTM 2000, and DESeq2 was used for gene expression analysis.

## Results

### Imbalances of ferroptosis and pyroptosis involved in the development of psoriasis

Psoriasis is an autoimmune disease mediated by overactive IL17 secreting type 17 T-cells (such as Th17 and Tc17) and defective negative regulatory networks (such as dysfunctional Tregs). Many researchers have focused on immune-related factors in the development of psoriasis, but haveignored the cell-fate transition of hyperproliferative keratinocytes themselves [31]. As part of this work, scRNA-seq analysis of psoriasis and control skin-derived cells were performed to identify the cell fate transition of nonimmune subsets (Additional file 1: Fig. S1A). Interestingly, compared with the control groups, the psoriatic skin-derived keratinocytes and fibroblasts showed a lower ferroptosis driver gene score (Additional file 8: Table S1 and Additional file 1: Fig. S1B). Some important ferroptosis driver genes, such as RPL8 [32], NCOA4 [33], and ALOXE3 [34], are expressed at low levels in the keratinocyte population in psoriasis. In addition, some critical ferroptosis suppressor genes, such as GPX4 [35], FTL, and FTH1 [36], are expressed at high levels in the keratinocyte population in psoriasis (Fig. 1A). These differences implied that ferroptosis is involved in the development of psoriasis. Keratinocyte cells were subdivided into stratum corneum (S.C, with high expression of KRT23, KRT80, SPRR2G, LCE3D, and CDSN), stratum granulosum, stratum spinosum (S.G & S.S, with high expression of FABP5, KRT10, and KRT1), and stratum basale (S.B, with high expression of MKI67, KRT14, and KRT5) (Fig. 1B) [37]. Psoriasis skin-derived S.B cells expressed GPX4, FTL, and FTH1 at high levels, whereas control skin-derived S.G & S.S cells expressed high levels of GPX4, FTH1, NR4A1, NFE2L2, and MT1G (Fig. 1C). Moreover, control skin-derived S.B and S.G & S.S cells expressed RPL8 at high levels, and control skin-derived S.C cells expressed high levels of NCOA4 and ALOXE3 (Additional file 1: Fig. S1C). These evidences indicated the ferroptosis-related pathways changed in psoriasis skin, and the S.B cells were given a combat advantage of ferroptosis, which is consistent with the phenotype of apoptosis-resistant psoriatic keratinocytes [38, 39]. A recent study showed that the partial index of active ferroptosis-related cell death in bulk psoriasis skin cells was higher than that in bulk normal skin cells[40]. In addition, we distinguished the differences in ferroptosis-related gene expression in different cell types, especially in immune cell types (Additional file 1: Fig. S1B) and S.G&S.S cells (Fig. 1C). It is noteworthy that inconsistency between the transcriptome and protein levels at single-cell level must be taken into consideration [28].

Deng et al. identified that Streptococcus cysteine protease SpeB virulence factor can trigger keratinocyte pyroptosis by cleaving GSDMA [41]. Therefore, it is proposed that pyroptosis also participates in the process of autoimmune skin diseases. Results showed that psoriasis skin-derived keratinocytes, fibroblasts, and endothelial cells (ECs) had a lower pyroptosis geneset score than control skin cells, with the exception of macrophages (Additional file 1: Fig. S1D and Fig. 1D). GSDMA and GSDMC were highly expressed in S.C cells, whereas GSDMB was highly expressed in S.B cells (Fig. 1E). Interestingly, compared with psoriasis skin-derived S.C cells, control skin cells highly expressed pyroptosis-related genes (CASP4, GSDMA, and GSDMC), suggesting that psoriasis skin-derived S.C cells were not sensitive to pyroptosis. In contrast, psoriasis skin-derived S.B cells expressed high levels of pyroptosis-related genes, such as CASP1, CAPS8, GSDMA, GSDMB, and GSDMD. Further, CASP3 was downregulated in S.C and S.G&S.S cells of psoriatic skin. Together, these data show that pyroptosis participates in the imbalance between the sensitivity of pyroptosis and apoptosis in different keratinocyte lineages (Fig. 1F). However, this biological phenotype requires further validation in future studies.

### GSDMC and GSDMD disrupted the keratinocyte differentiation of AD

**Fig. 2:**
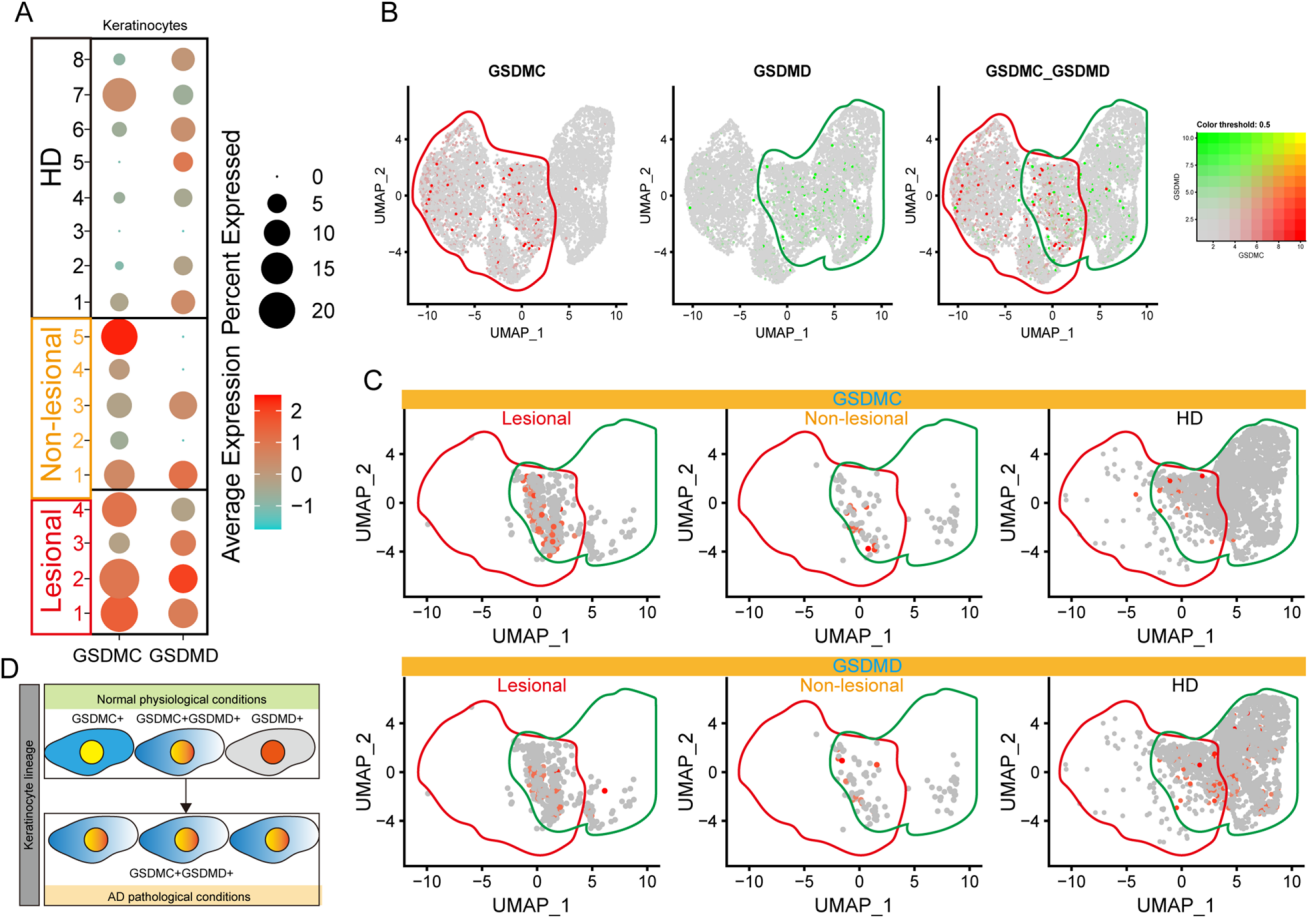
Novel pyroptosis-sensitive keratinocyte subsets in lesion AD. **(A)** Dot plot displays GSDMC and GSDMD mRNA expression in keratinocytes derived from AD lesional samples, AD non-lesional samples, and healthy samples. **(B)** Feature plots present expression of GSDMC and GSDMD in keratinocytes. **(C)** Feature plots display expression of GSDMC and GSDMD in keratinocytes during the disease. **(D)** Schematic depiction of the transition between normal keratinocytes and pyroptosis-sensitive keratinocytes

AD, a common inflammatory skin disease, is primarily characterized by a type-II immune response that leads to skin barrier damage [42]. To investigate potential cell death modalities, AD-related scRNA-seq datasets obtained from GSE147424 was analyzed. No major differences were observed in the ferroptosis suppressor gene score, ferroptosis driver gene score, and pyroptosis gene score in the fibroblast population, keratinocyte population, and EC population from healthy skin biopsy, lesional skin biopsy, and non-lesional biopsy (Additional file 2: Fig. S2A). However, GSDMC and GSDMD were highly expressed in lesional skin-derived keratinocytes (Fig. 2A). It is noteworthy that nuclear programmed death ligand 1 (nPD-L1) can upregulate GSDMC, whereas TNF-α-activated caspase-8 can cleave GSDMC, which will switch apoptosis to pyroptosis in cancer cells [43]. TNF-α has been shown to play a role in the pathogenesis of allergic inflammation in AD [44]. This evidence demonstrated that TNFα-Caspase8-GSDMC mediated pyroptosis may trigger keratinocyte death and inflammation. Keratinocytes were segregated into nine subtypes (Additional file 2: Fig. S2B, C). In addition, based on the expression patterns of GSDMC and GSDMD, keratinocytes were divided into three subpopulations: GSDMC^hi^GSDMD^low^, GSDMC^hi^GSDMD^hi^, and GSDMC^low^GSDMD^hi^ (Fig. 2B). Further analysis revealed that lesional skin-derived keratinocytes were located in the GSDMC^hi^GSDMD^hi^ subset (Fig. 2C). Accordingly, it is speculated that this unique transition of the pyroptosis-sensitive keratinocyte subpopulation may play a role in pyroptosisin AD.

### The pyroptosis and ferroptosis of epidermal melanocytes might drive vitiligo

**Fig. 3:**
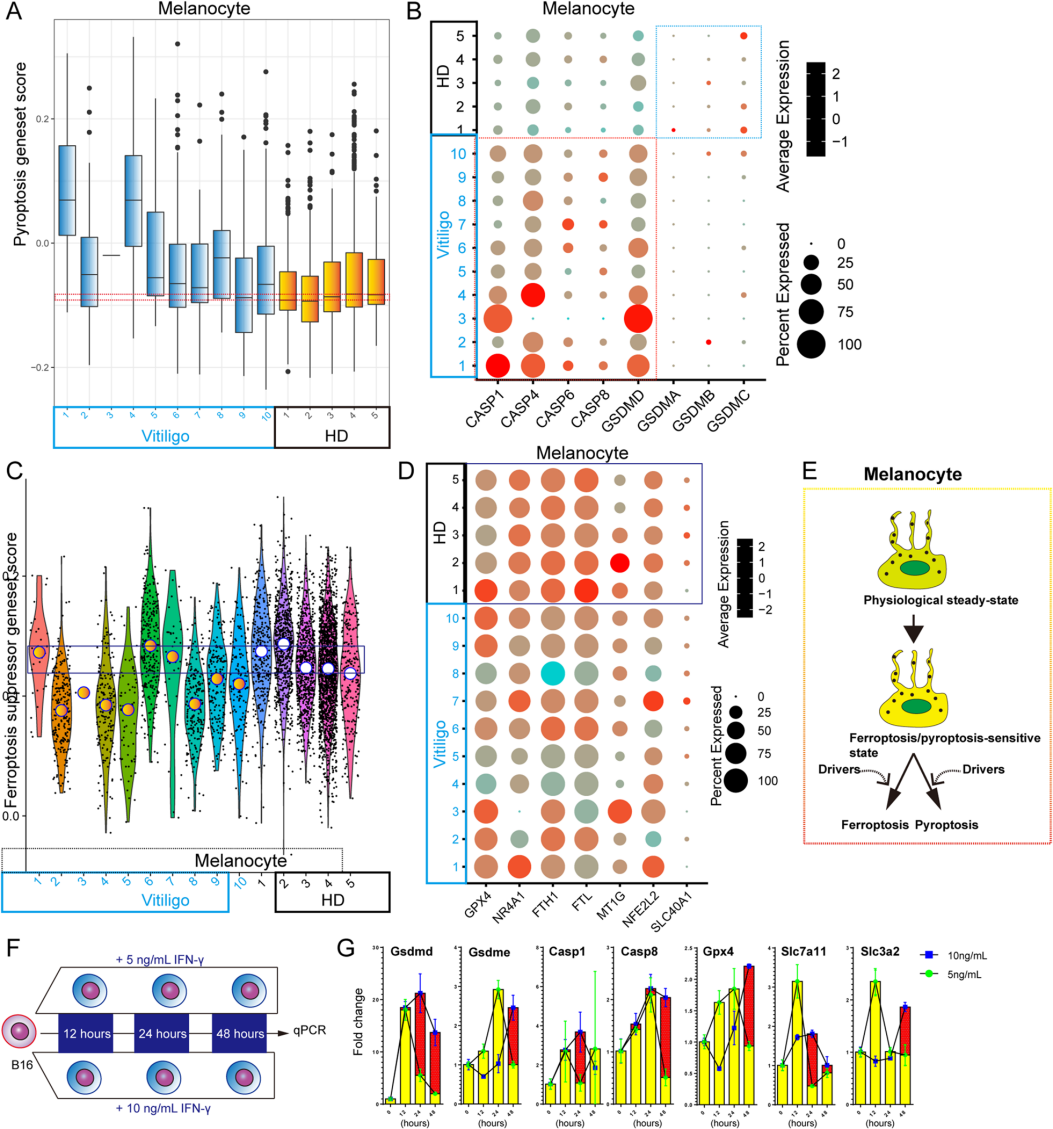
Pyroptosis and ferroptosis drive the melanocyte destruction. **(A)** Box plot showing pyroptosis geneset score of melanocytes within skin of patients with vitiligo and healthy donor skin. **(B)** Dot plot showing the expression levels of CASP1, CASP4, CASP6, CASP8, GSDMD, GSDMA, GSDMB, and GSDMC within melanocytes derived from vitiligo groups and control groups. **(C)** Quantification of ferroptosis suppressor geneset score within melanocytes derived from vitiligo groups and control groups. **(D)** Dot plot showing the expression levels of ferroptosis-resistant genes (GPX4, NR4A1, FTH1, FTL, MT1G, NFE2L2, and SLC40A1) within melanocytes. **(E)** Schematic depiction of cell fate of melanocytes under pathological condition

Vitiligo is caused by the loss of epidermal melanocytes due to the activity of autoreactive CD8^+^ T cells. To clarify the potential mechanism leading to the loss of epidermal melanocytes, we analyzed scRNA-seq datasets of skin-derived cells from healthy donors and patients with vitiligo (Additional file 3: Fig. S3A). The melanocytes were defined as signature genes (*DCT*, *TYRP1*, *PMEL*, and *MLANA*) (Additional file 3: Fig. S3B). Further analysis of melanocytes revealed strong pyroptosis responses existed in patients with vitiligo (Fig. 3A).Results demonstrated that CASP1, CASP4, CASP6, CASP8, and GSDMD expression was significantly upregulated in melanocytes of patients with vitiligo (Fig. 3B). As previously reported, CASP4, CASP8, and CASP1 are involved in GSDMD-dependent pyroptosis [45–47]. Caspase-6 promotes Z-DNA binding protein 1 (ZBP1)-mediated apoptosis, necroptosis and pyroptosis [48]. These results indicate that pyroptosis signaling is an important pathway in the development of vitiligo.

**Fig. 4:**
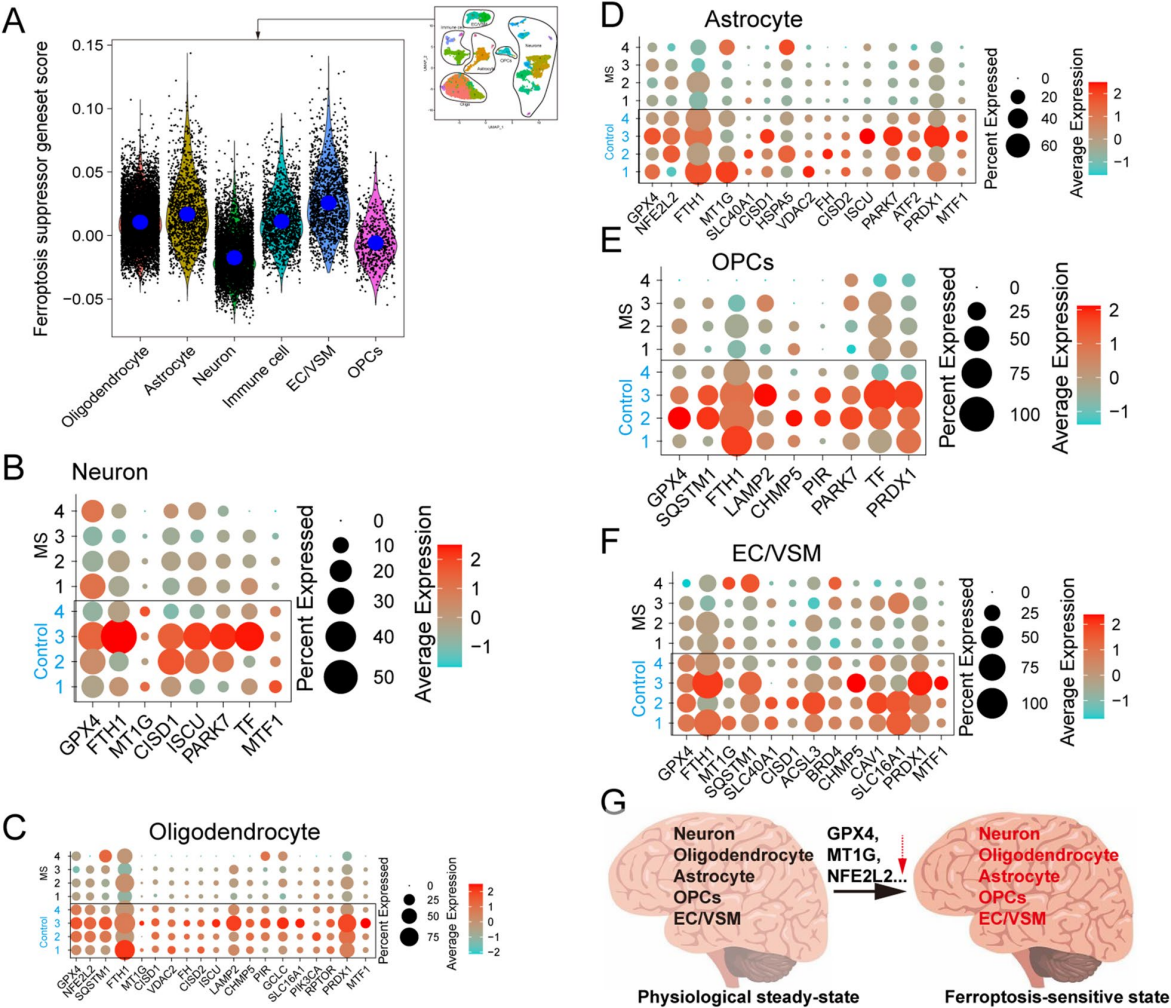
Ferroptosis-sensitivity was increased with decreasing expression of ferroptosis-resistant genes in brain cells of patients with MS. **(A)** Violin plot showing the ferroptosis suppressor geneset score in the different brain-derived cell subsets. **(B)** Dot plot showing the expression levels of GPX4, FTH1, MT1G, CISD, ISCU, PARK7, TF, and MTF1 within neurons of MS groups and control groups. Dot plot showing the expression levels of ferroptosis-resistant genes within oligodendrocytes **(C)**, astrocytes **(D)**, OPCs **(E)**, and EC/VSM **(F)** derived from MS groups and control groups. (G) Schematic depiction of ferroptosis-sensitive state under MS pathological condition

A ferroptosis-related analysis of melanocytes was conducted, in which only a small number of samples (1/10) showed a higher ferroptosis driver gene score than that of healthy donors (Additional file 3: Fig. S3C). Nevertheless, several important ferroptosis driver genes, such as ASCL4 and TFRC, were upregulated in the patient samples (Additional file 3: Fig. S3D). Acyl-CoA synthetase long-chain family member 4 (ACSL4), a critical component of ferroptosis, is enriched in cellular membranes with long polyunsaturated ω6 fatty acids and increases the sensitivity of target cells to ferroptosis [49]. TFRC overexpression increases the intracellular iron pool and enhances lipid peroxidation. Notably, we found that melanocytes of patients (7/10) had a lower ferroptosis suppressor gene score than that of healthy donors, indicating that these melanocytes were sensitive to ferroptosis (Fig. 3C). Healthy donor-derived melanocytes stably expressed key anti-ferroptosis genes at high levels, such as GPX4, NR4A1, FTH1, FTL, MT1G, and SLC40A1 (Fig. 4D). Our results confirmed that both pyroptosis and ferroptosis are involved in the loss of melanocytes during vitiligo development.

The IFN-γ signaling pathway is activated in melanocytes of patients with vitiligo [50]. Therefore, B16 cell line was used as a cell model to explore the effects of IFN-γ signaling-mediated ferroptosis and pyroptosis (Fig. 3F). IFN-γ-mediated pyroptosis depends on the IFN-γ concentration (Fig. 3G). In general, IFN-γ induces pyroptosis susceptibility based on upregulation of GSDMD, GSDME, CASP1, and CASP8. Interestingly, IFN-γ exposure did not significantly reduce the expression of GPX4, SLC7A11, or SLC3A2, implying that other factors are involved in the increasing ferroptosis-sensitive state of melanocytes in patients with vitiligo. Taken together, IFN-γ is a critical factor that drives the pyroptosis-sensitive state, but not ferroptosis.

### Ferroptosis participated in the pathology of multiple sclerosis (MS)

To investigate further the involvement of ferroptosis and pyroptosis in the pathology of autoimmune diseases, we compared the single-nucleus transcriptional profile of brain cells derived from MS patients and control individuals [51]. Although the two groups didn not show obvious differences in ferroptosis driver/suppressor gene scores and pyroptosis gene score (Additional file 4: Fig. S4), neurons had a lower ferroptosis suppressor gene score than other cell types (oligodendrocytes, astrocytes, immune cells, EC/VSM, and OPCs) (Fig. 4A). In addition, the neurons of MS patients expressed some important anti-ferroptosis genes (such as GPX4, FTH1, MT1G, and MTF1) at low levels (Fig. 4B). The same phenomenon was observed in some cell types, such as oligodendrocytes (GPX4, NFE2L2, FTH1, MT1G, SLC16A1, MTF1 etc.) (Fig. 4C), astrocytes (GPX4, NFE2L2, FTH1, MT1G, SLC40A1, MTF1 etc.) (Fig. 4D), OPCs (GPX4, SQSTM1, and FTH1) (Fig. 4E), and EC/VSM (GPX4, FTH1, MT1G, SQSTM1, SLC40A1, SLC16A1, and MTF1) (Fig. 4F). It has been observed that GPX4, as the most robust anti-ferrotposis functional gene, was commonly downregulated in MS patient-derived cell types, which convincingly demonstrated that ferroptosis is involved in the progression of MS. The downregulation of ferroptosis-resistant genes in MS brain cells leads to a tendency towards ferroptosis.

### Pyroptosis and ferroptosis were involved in the systemic sclerosis-associated interstitial lung disease

**Fig. 5:**
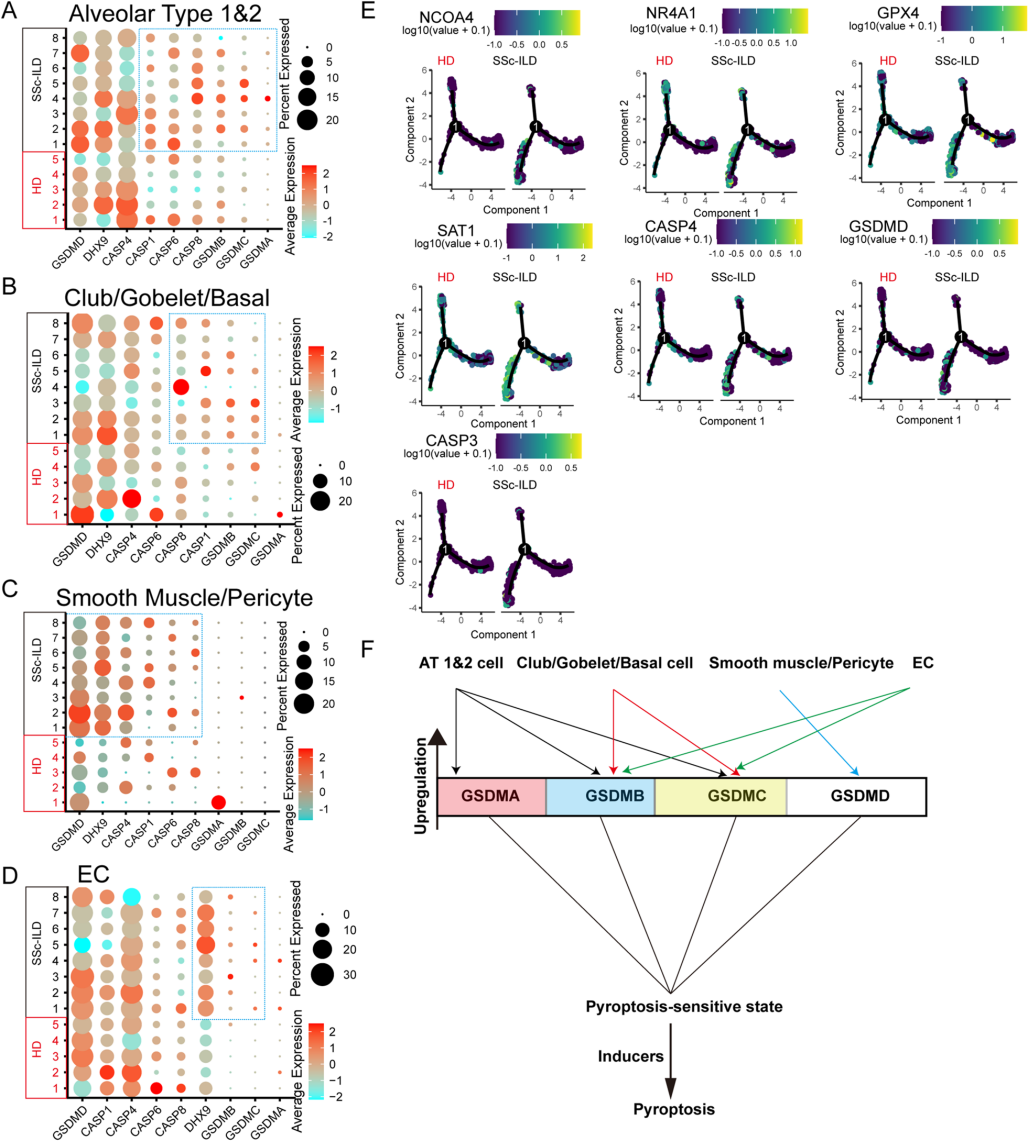
Pyroptosis involved in the pathological process of SSc-ILD. Expression levels of pyroptosis-related genes in different cell types, such as alveolar type 1&2 cells **(A)**, club/gobelet/basal cells **(B)**, smooth muscles/pericytes **(C)**, and ECs **(D)** across matched samples. **(E)** Selected important genes are shown in monocle2 produced pseudotime trajectory plot in different representative samples. **(F)** Schematic depiction of typical GSDM gene expression patterns within different cell types under pathological SSc-ILD condition

Systemic sclerosis (SSc) is an autoimmune disorder that can lead to interstitial lung disease (ILD). To explore the role of pyroptosis and ferroptosis in SSc-ILD, we analyzed the scRNA-seq dataset from GSE128169, which included eight SSc-ILD samples and five control samples. Analysis of pyroptosis-related driver genes in several major cell types revealed many significant changes between normal lungs and SSc-ILD samples. CASP1, CASP6, CASP8, GSDMB, GSDMC, and GSDMA were markedly upregulated in alveolar type 1&2 cells derived from SSc-ILD samples (Fig. 5A). Notably, group A Streptococcus (GAS) cysteine protease SpeB virulence factor can cleave GSDMA after Gln246, which triggers pyroptosis [41]. Upregulated GSDMA levels in patient with SSc may lead to pneumonia caused by Streptococcus pyogenes or other toxins. In addition, the club/gobelet/basal cells tended to upregulate the expression of CASP8, CASP1, GSDMB, and GSDMC in the SSc-ILD samples (Fig. 5B). Smooth muscle cells and pericytes in SSc-ILD lungs showed increased expression of GSDMD, DHX9, CASP4, CASP1, CASP6, and CASP8 compared to healthy cells (Fig. 5C). SSc-ILD lung-derived ECs exhibited high expression of DHX9, GSDMB, and GSDMC (Fig. 5D). Thus, increased GSDMB expression in alveolar type 1&2 cells, club/gobelet/basal cells, and ECs derived from SSc-ILD might increase the risk of pyroptosis mediated by the GZMA of autoactive CTLs.

To evaluate the role of ferroptosis in SSc-ILD, the ferroptosis suppressor/driver gene set score was identified and found no significant difference between healthy lungs and SSc-ILD lungs in several cell types (fibrobasts, alveolar type 1&2 cells, smooth muscle/pericytes, club/gobelet/basal cells, and ECs) (Additional file 5: Fig. S5A). Several studies have shown that fibroblasts play a key role in fibrotic ILD owing to abnormalities in aberrant extracellular matrix remodeling [52]. Accordingly, we analyzed the cell trajectory of fibroblasts, and identified two different branches with various effector gene expression patterns. Branch 1 expressed IL6 at high level, and branch 2 highly expressed profibrotic features, such as CXCL12 [53], VEGFA [54], IGF1 [55], and TGFB1 [56] (Additional file 5: Fig. S5B). This profibrotic branch mostly exists in SSc-ILD lungs, but not in healthy lungs, and expresses ferroptosis-related genes (ferroptosis-resistant genes: GPX4 and NR4A1 [57]; pro-ferroptotic genes: NCOA4 and SAT1 [58]); and pyroptosis drivers (CASP4 and GSDMD) (Fig. 5E). These results revealed that SSc-ILD profibrotic fibroblasts had high activity of ferroptosis and pyroptosis. Interestingly, SSc-ILD mast cells substantially expressed CASP4, CASP6, CASP8, DHX9, and GSDMD (Additional file 5: Fig. S5C), suggesting relationship between pyroptosis and mast cell degranulation.

### Ferroptosis was involved in the imbalance of intestinal microenvironment homeostasis in Crohn’s Disease

**Fig. 6:**
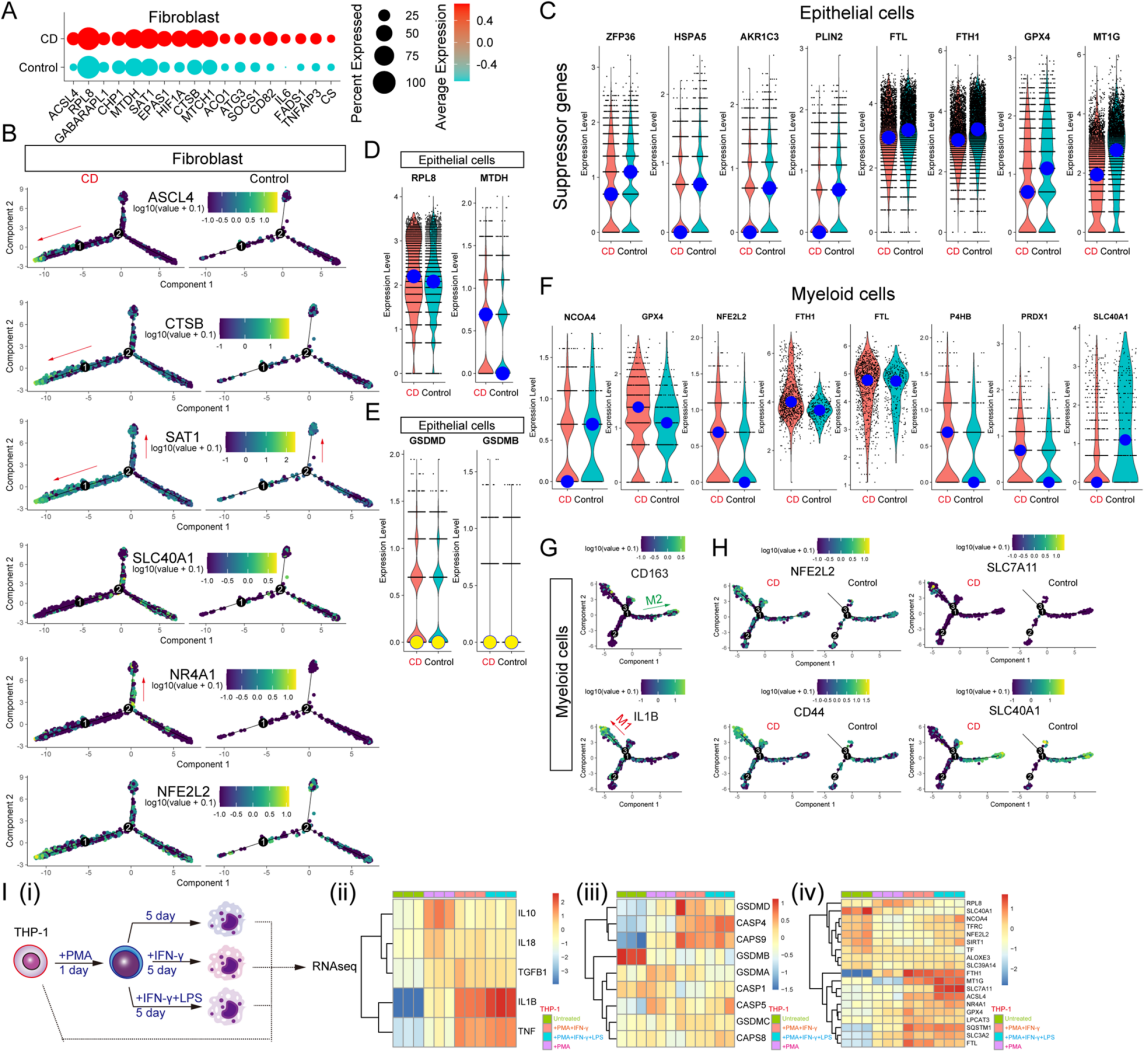
Aberrant ferroptosis and pyroptosis involved in the pathological process of CD. **(A)** Dot plot showing the expression levels of pro-ferroptosis genes within fibroblasts of CD samples and control samples. **(B)** Selected representative genes are shown in monocle2 produced pseudotime trajectory plots of fibroblasts under different conditions. Violin plots showing the expression levels of ferroptosis suppressor genes **(C)**, ferroptosis driver genes **(D)**, and pyroptosis-related genes **(E)** within epithelial cells of CD samples and control samples. **(F)** The expression levels of NCOA4, GPX4, NFE2L2, FTH1, FTL, P4HB, PRDX1, and SLC40A1 within myeloid cells of CD samples and control samples. **(G**, **H)** Selected important genes were shown in monocle2 produced pseudotime trajectory plot under different conditions. **(I)** **i**. The schematic of bulk RNA-seq of THP-1-derived cells. **ii**. Heatmap depicted gene expression levels of macrophage-related genes (M1: IL18, IL1B, and TNF; M2: IL10 and TGFB1). **iii**. Heatmap of expression levels of pyroptosis-related genes. **iv**. Heatmap shows expression levels of ferroptosis-related genes

Crohn’s disease (CD), a common form of inflammatory bowel disease (IBD), is characterized by irreversible aberrant immune responses [59]. Public scRNA-seq dataset of the terminal ileum of patients with CD and control donors was obtained from https://www.gutcellatlas.org/ (Additional file 6: Fig. S6A). First, we investigated the ferroptosis state of fibroblasts (Additional file 6: Fig. S6B), and confirmed that some proferroptosis genes, such as ACSL4, RPL8, SAT1, and CS [60], were upregulated (Fig. 6A). In addition, CD fibroblasts highly expressed some ferroptosis-resistant genes (such as FTH1, GPX4, NR4A1, NFE2L2, and FTL), but not others (such as MT1G and SLC40A1), indicating that pathological changes in fibroblast heterogeneity existed to some extent (Additional file 6: Fig. S6C). Consequently, the analysis of the cell trajectory of fibroblasts (Additional file 6: Fig. S6D) and revealed a unique differentiated cell state with ASCL4, CTSB, SAT1, and NFE2L2 expression at high levels (Fig. 6B). This differentiated cell state was consistent with SSc-ILD profibrotic fibroblast branch 2. Interestingly, this branch expressed SLC40A1 at low levels. These results highlighted that ferrotposis affected the balance of fibroblast differentiation. However there were no obvious differences in the pyroptosis-related genes between CD patient-derived fibroblasts and normal cells (Additional file 6: Fig. S6E, F).

The expression of multiple ferroptosis suppressors and driver genes in the epithelial cells (Fig. 6C, D) showed that epithelial cells derived from patients with CD expressed anti-ferroptosis genes (ZFP36 [61], HSPA5 [62], AKR1C3 [63], PLIN2 [64], FTL, FTH1, GPX4, and MT1G [65]) at lower levels than those of control cells, while pro-ferroptosis gene levels (RPL8 and MTDH [66]) were higher than those control cells. However, no clear evidence of upregulating pyroptosis drivers was identified (Additional file 6: Fig. S6G and Fig. 6E).

Next, it was necessary to investigatewhether pyroptosis and ferroptosis are involved in pathological myeloid differentiation. Myeloid cells in CD patients highly express anti-ferroptosis genes, such as GPX4, NFE2L2, FTH1, FTL, P4HB [67], and PRDX1 [68]. Moreover, compared to healthy myeloid cells, these myeloid cells expressed NCOA4 at low levels. Interestingly, SLC40A1 was highly expressed in healthy donor-derived myeloid cells (Fig. 6F). Cell trajectory analysis showed that macrophage M1 and M2 polarizations both existed in myeloid cells (Fig. 6G and Additional file 6: Fig. S6J). In addition, myeloid cells of patients with CD trended to polarize into the ferroptosis-resistant M1 state (Fig. 6H). SLC40A1 may also play a critical role in the maintaining of M2 state. SLC40A1 is highly expressed in tumor-associated macrophages, which suppresses the production of IL-1β [69], and is consistent with low IL-1β expression in control myeloid cells.

To identify the ferroptosis/pyroptosis patterns of macrophages under inflammatory conditions, as described CD inflammatory microenvironment, PMA, IFN-γ, and LPS were added to induce the differentiation of active macrophages and M1 macrophages (Fig. 6I(i)). Upon the PMA stimulation, THP-1 cells were induced into an activated state by secreting both M1 cytokines (IL18, IL1B, and TNF) and M2 cytokines (IL10 and TGFB1) (Fig. 6I(ii)). The cells were then differentiated into strong M1 states under IFN-γ and/or LPS culture conditions. Interestingly, with terminal M1 differentiation, THP-1-derived cells were more susceptible to pyroptosis owing to the upregulated expression of GSDMD, CASP4, CASP9, GSDMA, CASP5, GSDMC, and CASP8, but not GSDMB (Fig. 6I(iii)). The ferroptosis-related gene expression patterns of THP-1-derived M1-like cells were consistent with those of the above patterns of CD-related macrophages at scRNA-seq levels, such as SLC40A1 (Fig. 6I(iv)). THP-1-derived M1-like cells showed increased anti-ferroptotic activity (GPX4, NR4A1, FTH1, MT1G, SLC7A11, SLC3A2, and FTL) and decreased expression of NCOA4 and ALOXE3. Together, these results suggest that inflammatory conditions could drive the reversal of the sensitivity to ferroptosis and pyroptosis, and these properties could be targeted for treatment.

### The prominent role of ferroptosis in experimental autoimmune orchitis

We elucidated the roles of ferroptosis and pyroptosis in six human autoimmune diseases. Next scRNA-seq dataset analysis of experimental autoimmune orchitis (EAO) (Additional file 7: Fig. S7A, B), which is a widely used as the mouse model of testicular inflammation [70], was conducted. Compared with other cell types, spermatids had a low ferroptosis suppressor gene expression score, which indicated that spermatids were sensitive to ferrotposis (Additional file 7: Fig. S7C). Except for spermatogonia/sertoli cell cluster, control sample-derived spermatids, spermatocytes, and Leyding cells/immune cells showed a high ferroptosis suppressor geneset score (Additional file 7: Fig. S7D). There was no significant difference in the ferroptosis driver gene score and pyroptosis gene score between EAO and control samples or different clusters (Additional file 7: Fig. S7E–H). In addition, GPX4 was downregulated in EAO cells (Additional file 7: Fig. S7I). Specifically, spermatids, Leydig cells/immune cells, spermatocytes, and spermatigonia/sortoli cells in EAO trended to have reduced Gpx4 expression (Additional file 7: Fig. S7J). These results confirmed that testicular cells were sensitive to ferroptosis under EAO conditions.

## Discussion

The present study showed that in psoriasis, AD, vitiligo, MS, SSc-ILD, CD, and EAO, ferroptosis and pyroptosis act as disrupters with aberrant expression patterns. S.C cells of patients with psoriasis presented a cell death (ferroptosis/apoptosis/pyroptosis)-resistant pattern at the mRNA level. Meanwhile, S.G&S.S cells of patients with psoriasis possessed intensified resistance to apoptosis and pyroptosis, but were susceptible to ferroptosis. Our findings were consistent with those of a recent study [40], however, the roles of ferroptosis and pyroptosis in certain cell types of patients with psoriasis at the single cell level were highlighted in this work. Thus, our results provide valuable information to illustrate that some cell death-related drugs should be treated with great caution due to differences in cell death and changes in cell death sensitivity. Under AD pathological conditions, the keratinocyte lineage exhibits a unique and susceptible pyroptosis pattern. These keratinocytes tend to express both GSDMC and GSDMD, which might increase the sensitivity of pyroptosis. The high expression of GSDMC, suggests that inhibition of the TNFα-Caspase8 pathway may reduce the harmful effect of GSDMC-triggered pyroptosis [43]. Similarly, it may be helpful to reduce the risk of exposure to pathogens or other triggers of caspase-1 [71]. The pathogenesis of vitiligo is very clear [50, 72]. Epidermal melanocytes of patients with vitiligo have a remarkable features of pro-pyroptosis and pro-ferroptosis. A recent report has shown that ferroptosis is involved in the pathogenesis of melanocyte destruction in vitiligo [73], which was consistent with our results, and confirmed the reliability of our analysis methods. Some researchers have made an assumption without direct evidence that pyroptosis is involved in the pathogenesis of melanocyte destruction [74]. However, our results provide tangible evidence of pyroptosis-driven melanocyte destruction. The upregulation of CASP1/4 and GSDMD may drive canonical and non-canonical GSDMD-dependent pyroptosis of melanocytes. These findings collectively indicate that ferroptosis and pyroptosis were the important inducement of autoimmune skin diseases. What’s more, intestinal epithelial cells are ferroptosis-sensitive.

Compared with the known pyroptosis-driven melanocyte destruction in patients with vitiligo, pyroptosis-driven brain cell destruction was weak in MS. However, a pyroptosis-sensitive trend was detected (Additional file 4: Fig. S4). Indeed, GSDMD-mediated pyroptosis is involved in neuroinflammation of experimental autoimmune encephalomyelitis (EAE) model, which is initiated by peripheral myeloid cells [75]. Previous research has shown that GPX4 expression is decreased in the brains of patients with MS [76], which is consistent with our findings. Furthermore, this current study clarified which cell types were ferroptosis-sensitive at the single cell level, providing guidance on the selection of MS therapeutic strategies. Some researchers have noticed that pyroptosis may take part in the pathological alteration of pulmonary ECs in SSc [77]. Our results indicated that upregulation of GSDMB and GSDMC in SSc pulmonary ECs increased pyroptosis sensitivity. Different SSc pulmonary cell types upregulate different GSDM genes, such as GSDMA/B/C in AT1&2 cells, GSDMB/C in club/gobelet/basal cells, and GSDMD in smooth muscles/pericytes. Abnormal pyroptosis is linked to pathological transition of SSc-ILD and can be treated as a useful target. Macrophage-initiated pyroptosis is involved in the inflammatory response to CD. Pyroptosis-related features were not obvious in this work, while ferroptosis profoundly affected the balance of physiological and pathological fibroblasts, or M1 and M2 macrophages [78]. Thus, ferroptosis is involved in the pathological progression of EAO.

The ROS not only induece ferroptosis but also pyroptosis [79, 80], which points to the application of antioxidants in the treatment of some autoimmune diseases. Conventional antioxidant therapies have been proven to be less effective for several reasons, such as their inability to cross the blood–brain barrier, poor structural stability, low ROS and RNS scavenging activity, and low durability in vivo [81]. Nanomaterial antioxidants are good choice to solve these difficult problems, and several effective therapeutic nanomaterial antioxidants have been developed, such as two-dimensional (2D) transition-metal dichalcogenide (TMD) nanosheets [81], triapazamine-loaded hollow mesoporous bilirubin nanoparticles (HMBRN) [82], Fe3O4 @ TAn nanoflowers [83], monodispersed hydrophilic carbohydrate-derived nanoparticles (C-NP) [84], Pt-iNOS@ZIF nanoreactors [85], and ceria nanoparticles [86–88]. A recent study reported that an orally administered antioxidant nanoplatform based on simulated gastric fluid (SGF)-stabilized titanium carbide MXene nanosheets (Ti3C2 NSs) could be used to treat inflammatory bowel disease [89]. Nanoparticles can also be modified to load dexamethasone and antioxidants, which integrate ROS scavenging and anti-inflammatory drug delivery [90]. Some nanomaterial antioxidants, such as nanoenzyme-reinforced injectable hydrogel [91] and CIP-loaded and ceria-decorated polymer vesicles (CIP-Ceria-PVs) [92], have wide application potential in skin-related diseases. Mitochondria also participates in apoptosis via lipid peroxidation [93]. Many mitochondria-targeted ROS scavengers, such as mitoquinone (MitoQ) [94], SkQ1 [95], and melatonin [96] can prevent mitochondrial ROS formation and inhibit ferropotosis. Our results provide a theoretical foundation for the use of antioxidants based on ferropotosis and pyroptosis in autoimmune diseases.

Many studies on non-apoptotic regulated cell death (RCD) in tumor immunotherapy, including autophagy, ferroptosis, pyroptosis, and necroptosis, highlights the importance of research on non-apoptotic cell death mechanisms, and also indicate that these complex interactions can flow into several core molecular mechanisms [97]. Various microbial pathogen components and autoinflammatory factors also trigger these core molecular mechanisms, such as the CASP family/Granzyme-GSDM family axis and SLC7A11-GPX4 axis [98]. For example, *Talaromyces marneffei* can activate pyroptosis mediated by AIM2-caspase-1/-4-GSDMD in hepatocytes [99]. Moreover, some researchers have begun to pay attention to pyroptosis and ferroptosis in autoimmune diseases and drugs toxicity [100–104]. Our results provide pyroptosis/ferroptosis-associated signatures in several autoimmune diseases at the single-cell level, which provide extremely precise target information for the application of novel therapeutic approaches.

Taken together, the data presented herein strongly indicate that pyroptosis and ferroptosis are involved in autoimmune diseases (Table 2). scRNA-seq analysis was applied to understand the potential of programmed cell death in target cell types under pathological conditions. Our partial results were consistent with previous studies, demonstrating the reliability of the research methods adopted. In addition, the pattern and extent of ferroptosis and pyroptosis involvement in some autoimmune diseases determine the drugs that can be adopted to prevent uncontrolled cell death and inflammation. Notably, we found that IFN-γ is a key factor in increasing the sensitivity of pyroptosis, which provides a novel view for the role of IFN-γ-triggered pyroptosis in autoimmune diseases. Therapeutic strategies designed to inhibit the accumulation of ferroptosis/pyroptosis-sensitive target cells may improve clinical responses to autoimmune diseases. Thus, further studies based on in vivo experiments are required to screen for suitable drugs.

**Table 2:** Summary of ferroptosis and pyroptosis in autoimmune diseases

Disease type	Organs/tissues	Cell subset	Feature geneset (↑↓)	Sensitivity to ferroptosis/ pyroptosis (↑↓)
Vitiligo	Skin	Epidermal melanocytes	↑ Pyroptosis geneset (such as CASP1, CASP4, CASP6, CASP8, and GSDMD)	↑ Pyroptosis
			↓ Ferroptosis suppressor geneset (such as GPX4, NR4A1, FTH1, FTL, MT1G, NFE2L2, and SLC40A1)	↑ Ferroptosis
Psoriasis	Skin	Keratinocytes-S.C	↑ NR4A1, NFE2L2, MT1G	↓ Ferroptosis
			↓ CASP3, GSDMA, GSDMC	↓ Pyroptosis
		Keratinocytes-S.G&S.S	↓ GPX4, FTH1, NR4A1, NFE2L2, MT1G	↑ Ferroptosis
			↓ CASP1, CASP4, GSDMA	↓ Pyroptosis
		Keratinocytes-S.B	↑ GPX4, FTL, FTH1	↓ Ferroptosis
			↑ CASP1, CASP8, GSDMA, GSDMB, GSDMD	↑ Pyroptosis
AD	Skin	Keratinocytes	↑ GSDMC, GSDMD	↑ Pyroptosis
MS	Brain	Neuron	↓ GPX4, FTH1, and MT1G	↑Ferroptosis
		Oligodendrocyte	↓ GPX4, NFE2L2, SQSTM1, FTH1, and MT1G	↑Ferroptosis
		Astrocyte	↓ GPX4, NFE2L2, FTH1, MT1G, and SLC40A1	↑Ferroptosis
		OPCs	↓ GPX4, SQSTM1, and FTH1	↑Ferroptosis
		EC/VSM	↓ GPX4, FTH1, MT1G, SQSTM1, and SLC40A1	↑Ferroptosis
SSc-ILD	Lung	Alveolar type 1&2	↑ CASP1, CASP6, CASP8, GSDMB, GSDMC, and GSDMA	↑Pyroptosis
		Club/Gobelet/Basal	↑ CASP8, CASP1, GSDMB, and GSDMC	↑Pyroptosis
		Smooth Muscle/Pericyte	↑ CASP1, CASP4, CASP6, CASP8, and GSDMD	↑Pyroptosis
		EC	↑ GSDMB and GSDMC	↑Pyroptosis
		Mast cell	↑ CASP4, CASP6, CASP8, and GSDMD	↑Pyroptosis
CD	Terminal ileum	Epithelial cells	↓ GPX4, MT1G, FTL, and FTH1	↑Ferroptosis
EAO	Testes	Spermatids	↓ Ferroptosis suppressor geneset (such as GPX4)	↑Ferroptosis
		Spermatocytes	↓ Ferroptosis suppressor geneset (such as GPX4)	↑Ferroptosis
		Leydig cells/immune cells	↓ Ferroptosis suppressor geneset (such as GPX4)	↑Ferroptosis
		Spermatogonia/Sertoli cells	↓ Ferroptosis suppressor geneset (such as GPX4)	↑Ferroptosis

## Supplementary Information

**Additional file 1: Figure S1.** The expression patterns of ferroptosis and pyroptosis-related genes in different cell types derived from kin of psoriasis patients and healthy donor skin. (**A**) UMAP plot of cells derived from the skin of patients with psoriasis and healthy donors. (**B**) Quantification of ferroptosis driver geneset score and ferroptosis suppressor geneset score in different cell types (keratinocytes, fibroblasts, DCs, macrophages, and ECs) derived from the skin of patients with psoriasis and healthy donor. (**C**) Dot plot shows the expression levels of RPL8, NCOA4, and ALOXE3 in different keratinocyte subsets derived from psoriasis skin and healthy skin. (**D**) Quantification of pyroptosis geneset score in total cells and different subsets (fibroblasts, DCs, macrophages, and ECs) derived from the skin of patients with psoriasis and healthy donors.

**Additional file 2: Figure S2.** The expression patterns of ferroptosis and pyroptosis-related genes in different cell types of skin of patients with AD and healthy donor skin. (**A**) Quantification of ferroptosis suppressor geneset score and ferroptosis driver geneset score in different cell types (fibroblasts, keratinocytes, and ECs) under the disease condition. (**B**) Dot plot showing the expression of feature genes in different keratinocyte subsets. (**C)** UMAP plot of keratinocytes from skin of patients with AD and healthy donor skin.

**Additional file 3: Figure S3.** The state of ferroptosis driver in melanocytes. (**A**) UMAP plot of melanocytes from skin of patient with vitiligo and healthy skin. (**B**) Feature plot showing the expression level of DCT, TYRP1, PMEL, and MLANA. (**C**) Violin plots showing ferroptosis driver geneset score in different skin melanocytes. (**D**) Dot plot showing the expression level of ASCL4, NCOA4, LPCAT3, TF, and TFRC within melanocytes derived from vitiligo groups and control groups. (**F**) The schematic of qPCR of IFN-γ-treated B16 cells. (**G**) mGsdmd, Gsdme, Casp1, Casp8, Gpx4, Slc7a11, and Slc3a2 mRNA levels assessed by qPCR.

**Additional file 4: Figure S4.** Quantification of ferroptosis suppressor geneset score, ferroptosis driver geneset score, and pyroptosis geneset score within different cell types of MS groups and control groups.

**Additional file 5: Figure S5. (A**) The feature of ferroptosis within different cell types of patient samples and control samples. (**B**) Feature genes were shown in pseudotime trajectory plots. (**C**) Dot plot showing the expression levels of CASP1, CASP4, CASP6, CASP8, DHX9, GSDMD, GSDMB, GSDMC, and GSDMA within mast cells derived from patient samples and control samples.

**Additional file 6: Figure S6.** (**A**) UMAP plot of intestinal cells from healthy children and patients with CD. (**B**) Heatmap showing the relative mean expression levels of ferroptosis-related genes across fibroblasts derived from healthy children and patients with CD. (**C**) Dot plot showing the expression levels of ferroptosis-related genes within fibroblasts. (**D**) Pseudotime trajectory plot of fibroblasts. (**E**) Heatmap showing the relative mean expression of pyroptosis-related genes within fibroblasts derived from CD samples and control samples. (**F**) Violin plot showing the expression level of GSDMB and GSDMD within fibroblasts of patients with CD and healthy donors. (**G**) Heatmap showing the relative mean expression of pyroptosis-related genes within epithelial cells. (**H**) Violin plot showing the expression level of TGFB1 and IL1B within myeloid cells derived from CD samples and healthy samples.

**Additional file 7: Figure S7.** Decreasing GPX4 portended the ferroptosis involving EAO. **(A)** UMAP plot of testis cells from EAO models and control tissues. (**B**) Dot plot showing the feature genes of different major cell types. (**C**) Violin plot showing the quantification of ferroptosis suppressor geneset score and ferroptosis driver geneset score within testis cells. (D) Quantification of ferroptosis suppressor geneset score of different subsets under pathological or normal condition. (**E**) Quantification of ferroptosis driver geneset score within different cell types of EAO tissues and control tissues. (**F**) Box plot showing the quantification of pyroptosis geneset score in EAO tissues and control tissues. (**G**) Quantification of pyroptosis geneset score in different testis cell types. (**H**) Comparison of the quantification of pyroptosis geneset score in major cell types between EAO tissues and control tissues. (**I**) The expression level of Gpx4 in total testis cells derived from EAO samples or control samples. (**J**) Ridgeline plot showing Gpx4 expression level within different cell types of EAO samples or control samples.

**Additional file 8: Table S1.** The human geneset of ferrotosis driver.

**Additional file 9: Table S2.** The human geneset of ferrotosis suppressor.

**Additional file 10: Table S3.** The mouse geneset of ferrotosis driver.

**Additional file 11: Table S4.** The mouse geneset of ferrotosis suppressor.

## Data Availability

All data generated or analyzed in this study are included in this article. Other data that are relevant to this article are available from the corresponding author upon reasonable request.
