# Supplementary figures and images for: Evidence of pyroptosis and ferroptosis extensively involved in autoimmune diseases at the single-cell transcriptome level
Source: J Transl Med. 2022 Aug 12;20:363. doi: 10.1186/s12967-022-03566-6 (PMC9373312; doi:10.1186/s12967-022-03566-6)

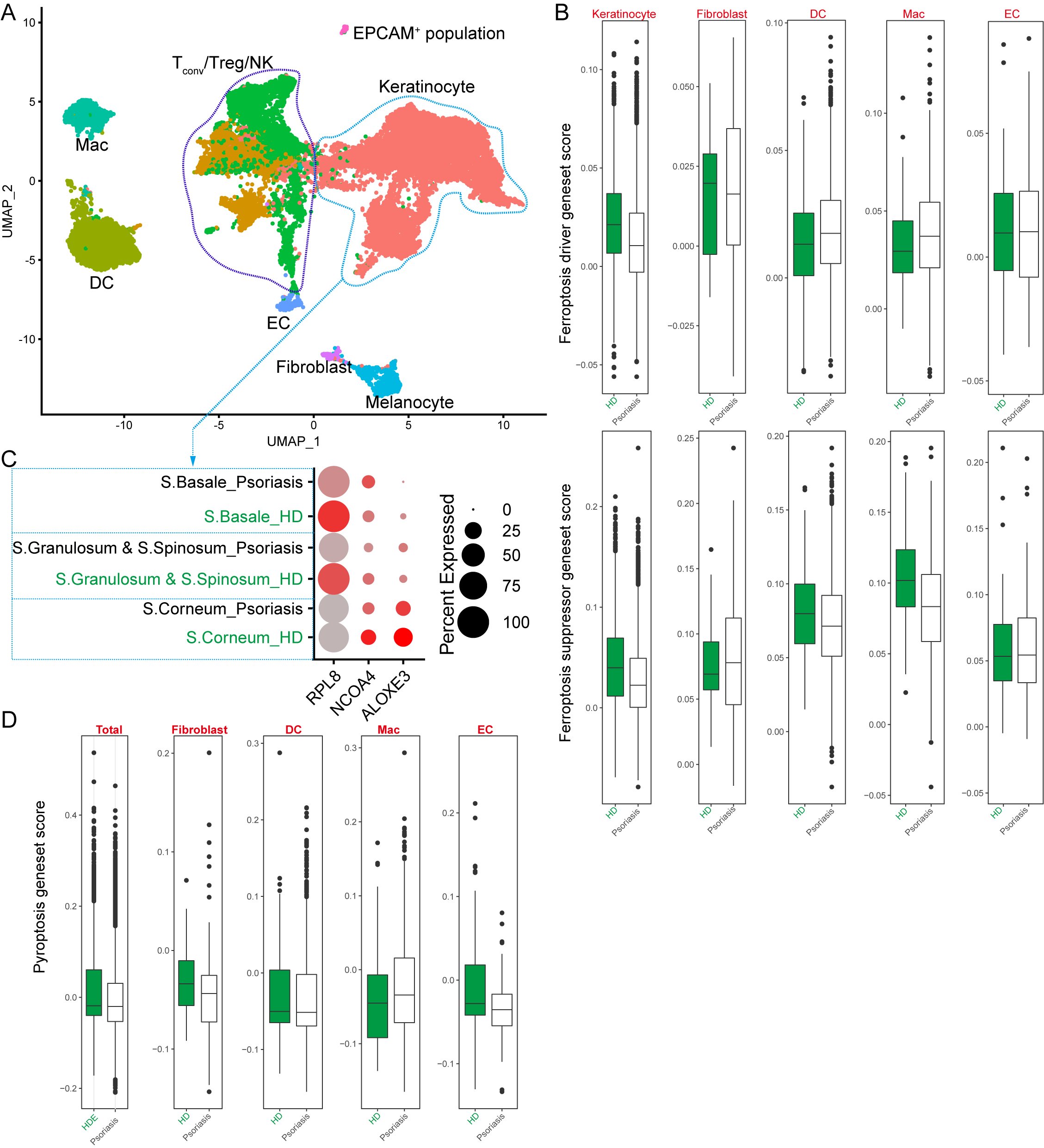

Supplement: Supplementary file 1 — Additional file 1: Figure S1. The expression patterns of ferroptosis and pyroptosis-related genes in different cell types derived from kin of psoriasis patients and healthy donor skin. (A) UMAP plot of cells derived from the skin of patients with psoriasis and healthy donors. (B) Quantification of ferroptosis driver geneset score and ferroptosis suppressor geneset score in different cell types (keratinocytes, fibroblasts, DCs, macrophages, and ECs) derived from the skin of patients with psoriasis and healthy donor. (C) Dot plot shows the expression levels of RPL8, NCOA4, and ALOXE3 in different keratinocyte subsets derived from psoriasis skin and healthy skin. (D) Quantification of pyroptosis geneset score in total cells and different subsets (fibroblasts, DCs, macrophages, and ECs) derived from the skin of patients with psoriasis and healthy donors. [file 12967_2022_3566_MOESM1_ESM.tif]

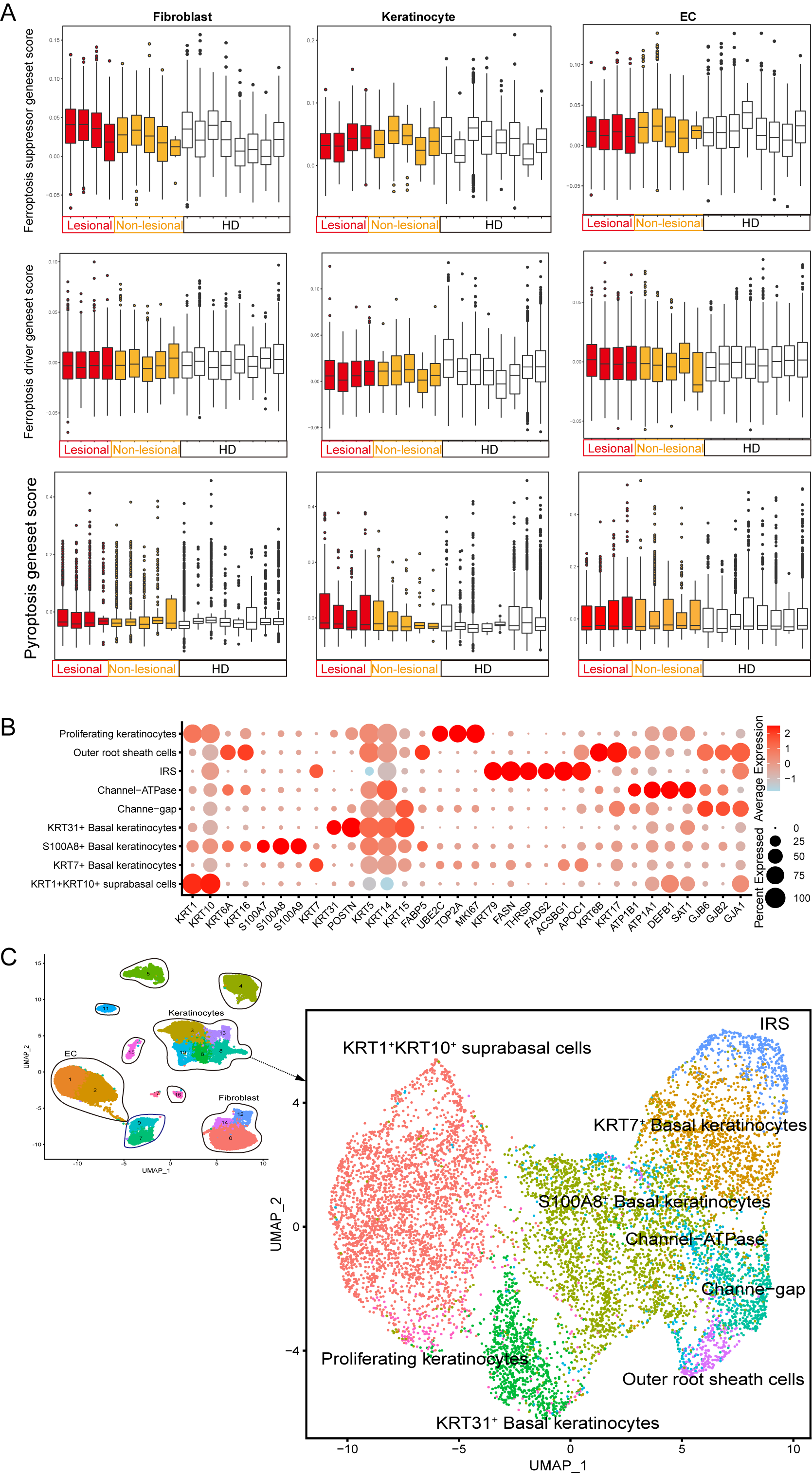

Supplement: Supplementary file 2 — Additional file 2: Figure S2. The expression patterns of ferroptosis and pyroptosis-related genes in different cell types of skin of patients with AD and healthy donor skin. (A) Quantification of ferroptosis suppressor geneset score and ferroptosis driver geneset score in different cell types (fibroblasts, keratinocytes, and ECs) under the disease condition. (B) Dot plot showing the expression of feature genes in different keratinocyte subsets. (C) UMAP plot of keratinocytes from skin of patients with AD and healthy donor skin. [file 12967_2022_3566_MOESM2_ESM.tif]

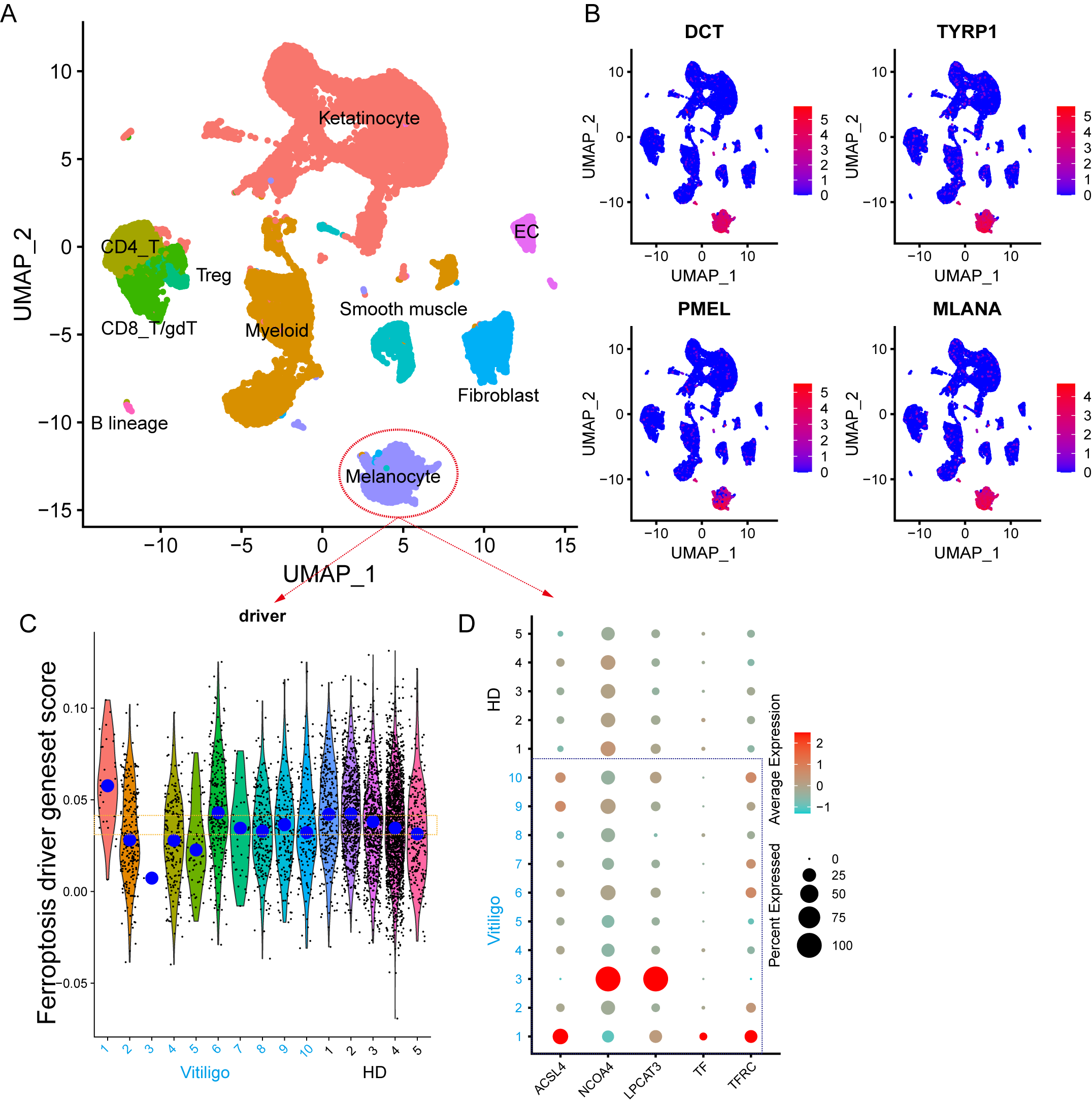

Supplement: Supplementary file 3 — Additional file 3: Figure S3. The state of ferroptosis driver in melanocytes. (A) UMAP plot of melanocytes from skin of patient with vitiligo and healthy skin. (B) Feature plot showing the expression level of DCT, TYRP1, PMEL, and MLANA. (C) Violin plots showing ferroptosis driver geneset score in different skin melanocytes. (D) Dot plot showing the expression level of ASCL4, NCOA4, LPCAT3, TF, and TFRC within melanocytes derived from vitiligo groups and control groups. (F) The schematic of qPCR of IFN-γ-treated B16 cells. (G) mGsdmd, Gsdme, Casp1, Casp8, Gpx4, Slc7a11, and Slc3a2 mRNA levels assessed by qPCR. [file 12967_2022_3566_MOESM3_ESM.tif]

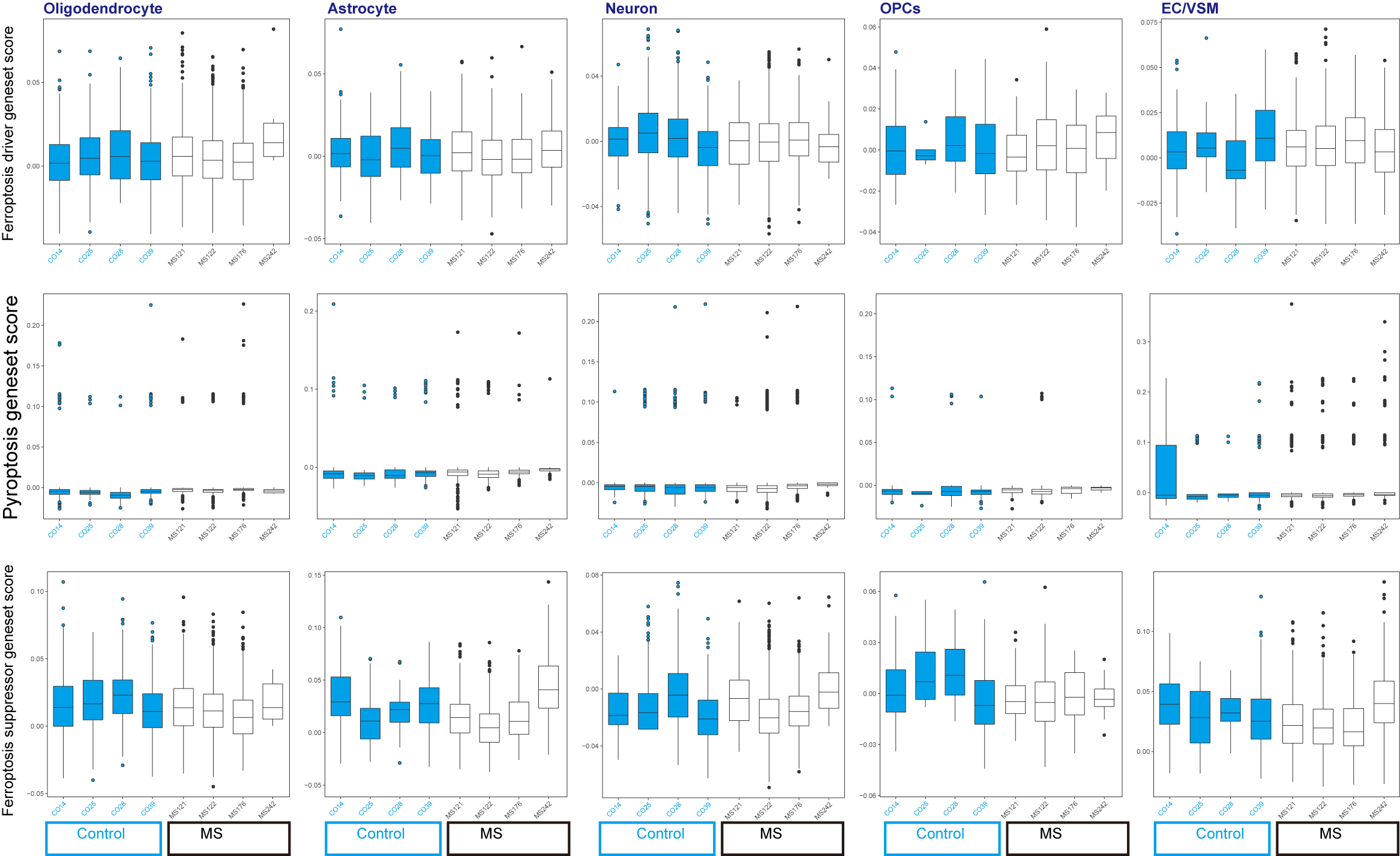

Supplement: Supplementary file 4 — Additional file 4: Figure S4. Quantification of ferroptosis suppressor geneset score, ferroptosis driver geneset score, and pyroptosis geneset score within different cell types of MS groups and control groups. [file 12967_2022_3566_MOESM4_ESM.tif]

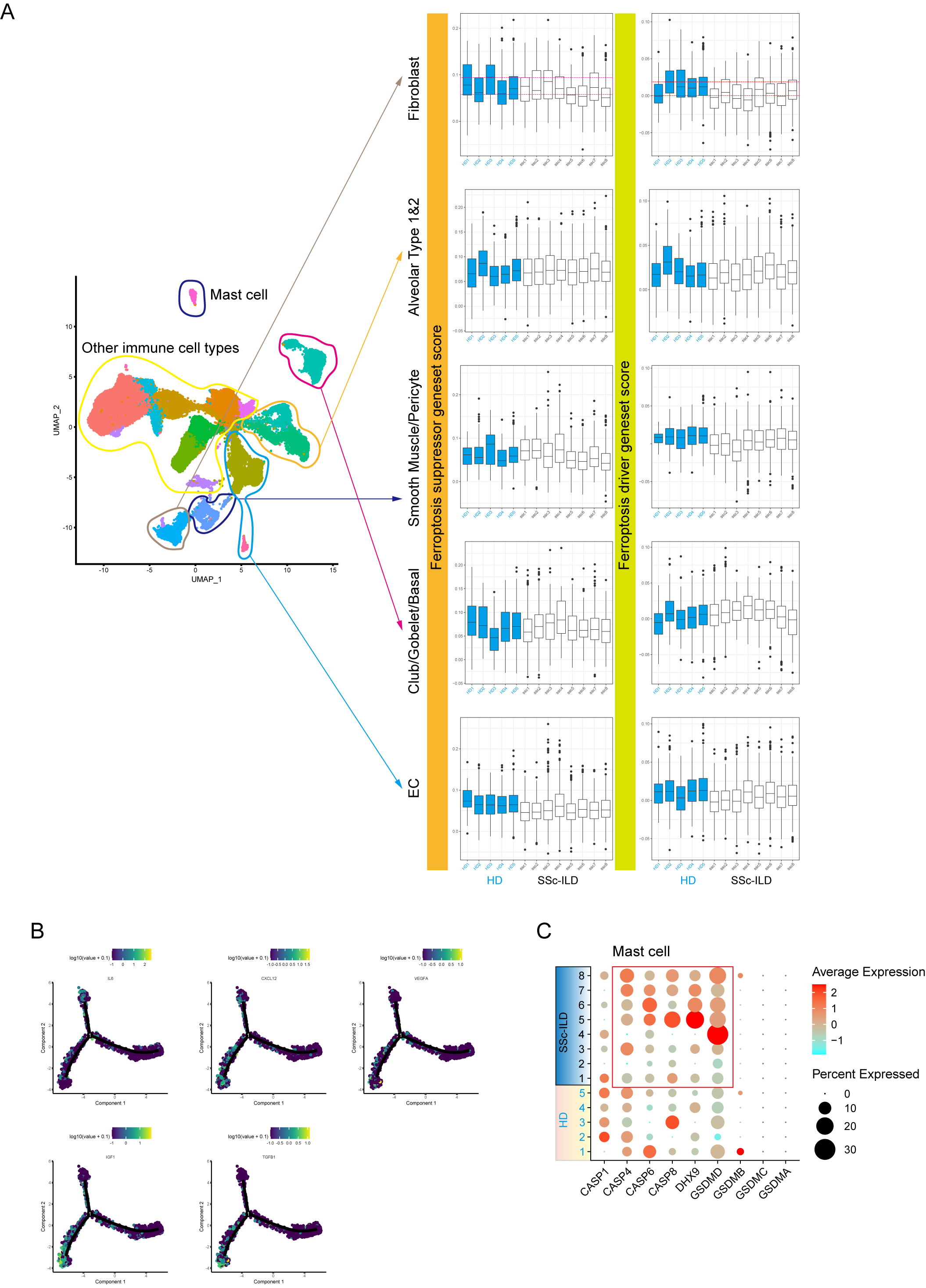

Supplement: Supplementary file 5 — Additional file 5: Figure S5. (A) The feature of ferroptosis within different cell types of patient samples and control samples. (B) Feature genes were shown in pseudotime trajectory plots. (C) Dot plot showing the expression levels of CASP1, CASP4, CASP6, CASP8, DHX9, GSDMD, GSDMB, GSDMC, and GSDMA within mast cells derived from patient samples and control samples. [file 12967_2022_3566_MOESM5_ESM.tif]

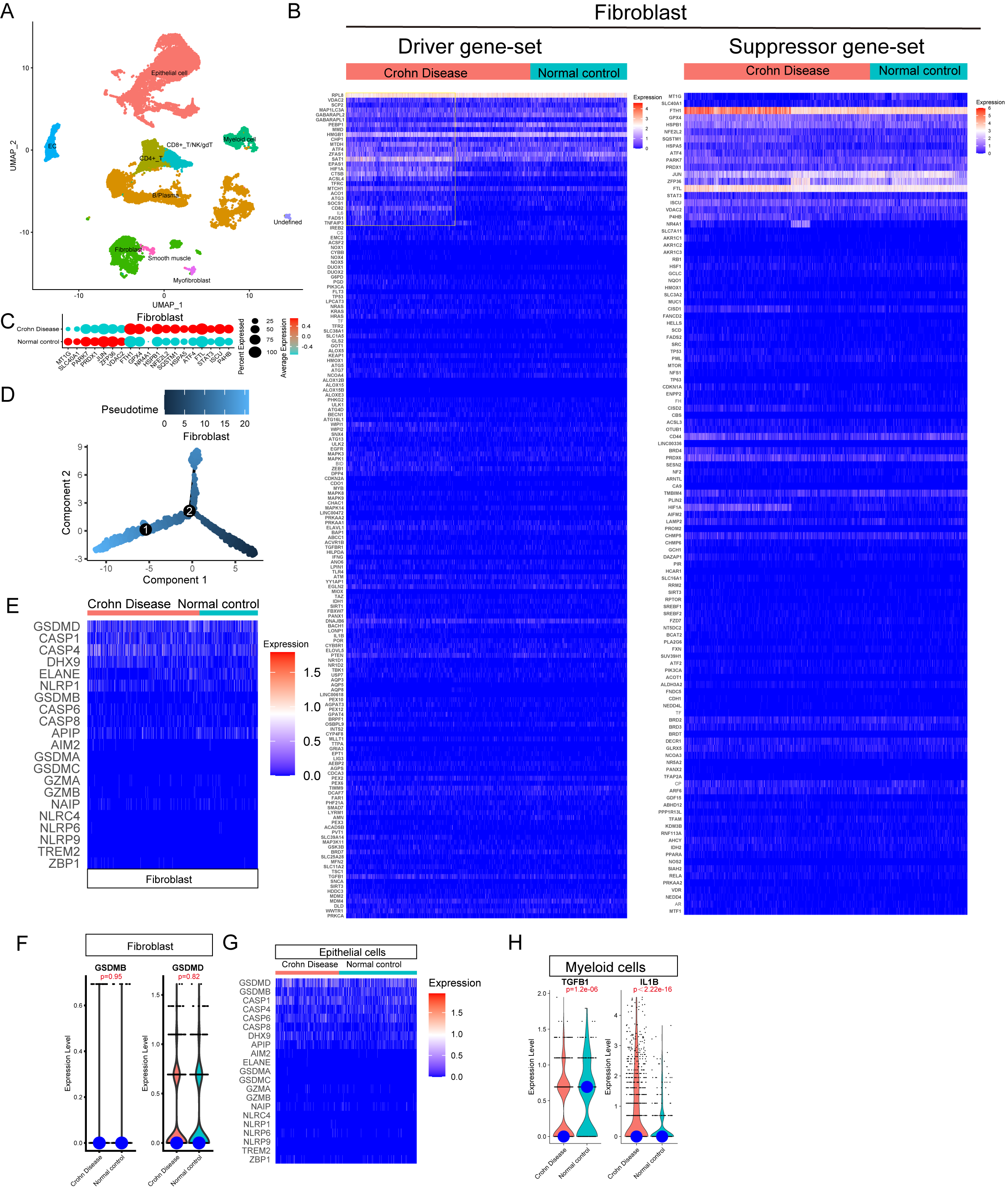

Supplement: Supplementary file 6 — Additional file 6: Figure S6. (A) UMAP plot of intestinal cells from healthy children and patients with CD. (B) Heatmap showing the relative mean expression levels of ferroptosis-related genes across fibroblasts derived from healthy children and patients with CD. (C) Dot plot showing the expression levels of ferroptosis-related genes within fibroblasts. (D) Pseudotime trajectory plot of fibroblasts. (E) Heatmap showing the relative mean expression of pyroptosis-related genes within fibroblasts derived from CD samples and control samples. (F) Violin plot showing the expression level of GSDMB and GSDMD within fibroblasts of patients with CD and healthy donors. (G) Heatmap showing the relative mean expression of pyroptosis-related genes within epithelial cells. (H) Violin plot showing the expression level of TGFB1 and IL1B within myeloid cells derived from CD samples and healthy samples. [file 12967_2022_3566_MOESM6_ESM.tif]

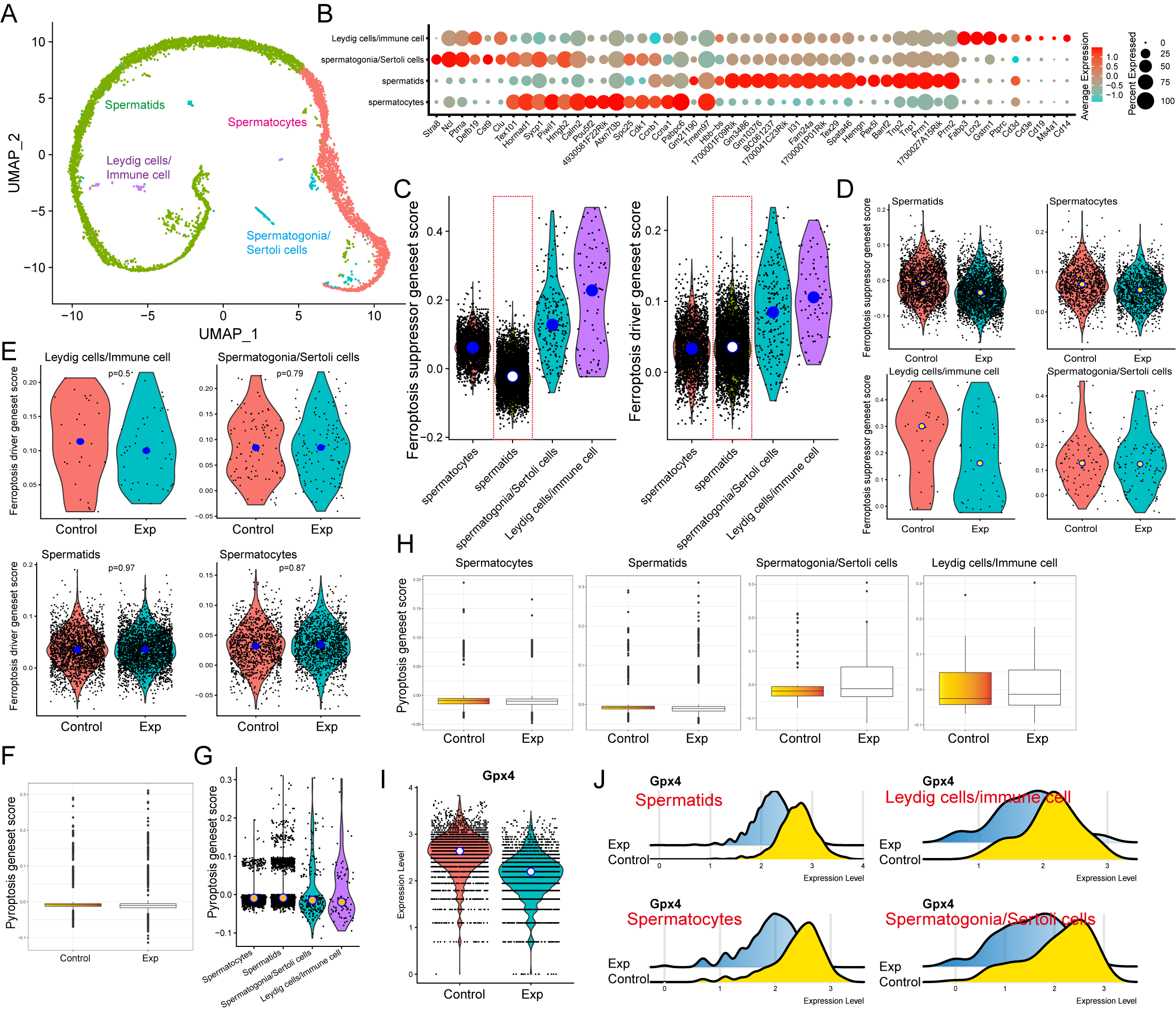

Supplement: Supplementary file 7 — Additional file 7: Figure S7. Decreasing GPX4 portended the ferroptosis involving EAO. (A) UMAP plot of testis cells from EAO models and control tissues. (B) Dot plot showing the feature genes of different major cell types. (C) Violin plot showing the quantification of ferroptosis suppressor geneset score and ferroptosis driver geneset score within testis cells. (D) Quantification of ferroptosis suppressor geneset score of different subsets under pathological or normal condition. (E) Quantification of ferroptosis driver geneset score within different cell types of EAO tissues and control tissues. (F) Box plot showing the quantification of pyroptosis geneset score in EAO tissues and control tissues. (G) Quantification of pyroptosis geneset score in different testis cell types. (H) Comparison of the quantification of pyroptosis geneset score in major cell types between EAO tissues and control tissues. (I) The expression level of Gpx4 in total testis cells derived from EAO samples or control samples. (J) Ridgeline plot showing Gpx4 expression level within different cell types of EAO samples or control samples. [file 12967_2022_3566_MOESM7_ESM.tif]
