# Supplementary material for: Evidence of pyroptosis and ferroptosis extensively involved in autoimmune diseases at the single-cell transcriptome level
Source: J Transl Med. 2022 Aug 12;20:363. doi: 10.1186/s12967-022-03566-6 (PMC9373312; doi:10.1186/s12967-022-03566-6)
Supplement: Supplementary file 8 — Additional file 8: Table S1. The human geneset of ferrotosis driver. [file 12967_2022_3566_MOESM8_ESM.docx]

Additional file Table S1. The human geneset of ferrotosis driver

| Ferrotosis driver genes | RPL8, IREB2, ATP5MC3, CS, EMC2, ACSF2, NOX1, CYBB, NOX3, NOX4, NOX5, DUOX1, DUOX2, G6PD, PGD, VDAC2, TP53, ACSL4, LPCAT3, NRAS, KRAS, HRAS, CARS1, KEAP1, HMOX1, ATG5, ATG7, NCOA4, TF, ALOX5, ALOX12, ALOX12B, ALOX15, ALOX15B, ALOXE3, PHKG2, SAT1, EGFR, MAPK3, MAPK1, ZEB1, DPP4, CDKN2A, PEBP1, SOCS1, CDO1, MYB, SLC1A5, CHAC1, LINC00472, GOT1, BECN1, PRKAA2, PRKAA1, ELAVL1, BAP1, ABCC1, MIR6852, ACVR1B, TGFBR1, IFNG, ANO6, HMGB1, TNFAIP3, ATF3, ATM, YY1AP1, EGLN2, MIOX, TAZ, MTDH, IDH1, FBXW7, PANX1, DNAJB6, LONP1, CD82, IL1B, POR, CYB5R1, ELOVL5, FADS1, FBW7, PTEN, IL6, miR-182-5p, miR-378a-3p, CTSB, ATF4, LINC00618, MT1DP, PEX10, AGPAT3, PEX12, CHP1, GPAT4, BRPF1, OSBPL9, INTS2, MMD, CYP4F8, MLLT1, TTPA, GRIA3, EPT1, POM121L12, LIG3, AEBP2, AGPS, CDCA3, PEX2, PEX6, TIMM9, DCAF7, LCE2C, FAR1, PHF21A, SMAD7, LYRM1, AMN, PEX3, MTCH1, SIRT1, ACADSB, PVT1, hsa_circ_0008367, GSK3B, MAPK8, BRD7, SLC25A28, SLC11A2, ZFAS1, SLC38A1, TSC1, TGFB1, SNCA, SIRT3, TFRC, CGAS, STING1, HDDC3, MIR761, MDM2, MDM4, MIR214, DLD, WWTR1, PRKCA |
| --- | --- |
