# Supplementary material for: Evidence of pyroptosis and ferroptosis extensively involved in autoimmune diseases at the single-cell transcriptome level
Source: J Transl Med. 2022 Aug 12;20:363. doi: 10.1186/s12967-022-03566-6 (PMC9373312; doi:10.1186/s12967-022-03566-6)
Supplement: Supplementary file 9 — Additional file 9: Table S2. The human geneset of ferrotosis suppressor. [file 12967_2022_3566_MOESM9_ESM.docx]

Additional file Table S2. The human geneset of ferrotosis suppressor

| Ferrotosis suppressor genes | SLC7A11, GPX4, AKR1C1, AKR1C2, AKR1C3, RB1, HSPB1, HSF1, NFE2L2, SQSTM1, NQO1, HMOX1, FTH1, MUC1, MT1G, SLC40A1, CISD1, HSPA5, ATF4, TP53, HELLS, SCD, FADS2, SRC, STAT3, PML, NFS1, TP63, CDKN1A, MIR137, VDAC2, FH, CISD2, MIR9-1, MIR9-2, MIR9-3, CBS, ISCU, ACSL3, OTUB1, CD44, LINC00336, BRD4, PRDX6, MIR17, SESN2, NF2, ARNTL, HIF1A, JUN, CA9, TMBIM4, PLIN2, AIFM2, LAMP2, ZFP36, PROM2, CHMP5, CHMP6, CAV1, GCH1, SIRT3, PIR, GCLC, HCAR1, SLC16A1, RRM2, NR4A1, PIK3CA, RPTOR, SREBF1, SREBF2, FZD7, P4HB, BCAT2, PLA2G6, MIR424, PARK7, FXN, SUV39H1, ATF2, STK11, FNDC5, CircIL4R, CDH1, MIR214, NEDD4L, TF, FTMT, BRD2, BRD3, BRDT, DECR1, GLRX5, NCOA3, NR5A2, MTOR, PANX2, RHEBP1, TFAP2A, CP, ARF6, GDF15, ABHD12, TFAM, KDM3B, RNF113A , AHCY, circ-TTBK2, MIR522, IDH2, PPARA, SIAH2, PRKAA2, NEDD4, PRDX1, AR, MTF1 |
| --- | --- |
