# Supplementary material for: Evidence of pyroptosis and ferroptosis extensively involved in autoimmune diseases at the single-cell transcriptome level
Source: J Transl Med. 2022 Aug 12;20:363. doi: 10.1186/s12967-022-03566-6 (PMC9373312; doi:10.1186/s12967-022-03566-6)
Supplement: Supplementary file 10 — Additional file 10: Table S3. The mouse geneset of ferrotosis driver. [file 12967_2022_3566_MOESM10_ESM.docx]

Additional file Table S3. The mouse geneset of ferrotosis driver

| Ferrotosis driver genes | Pik3ca, Flt3, Scp2, Tp53, Tf, Tfrc, Tfr2, Slc38a1, Slc1a5, Gls2, Got1, Alox5, Keap1, Hmox1, Atg5, Atg7, Aco1, Ireb2, G6pdx, Ulk1, Atg3, Atg4d, Becn1, Map1lc3a, Gabarapl2, Gabarapl1, Atg16l1, Wipi1, Wipi2, Snx4, Atg13, Ulk2, Ncoa4, Sat1, Acsl4, Lpcat3, Alox15, Bid, Dpp4, Cdkn2a, Pebp1, Mapk14, Elavl1, Epas1, Hilpda, Hif1a, Alox12, Ifng, Ano6, Lpin1, Hmgb1, Yy1ap1, Egln2, Miox, Taz, Mtdh, Sirt1, Fbxw7, Panx1, Dnajb6, Bach1, Il1b, Por, Nr1d1, Nr1d2, Tbk1, Il6, Ctsb, Atf4, Pex10, Lig3, Zeb1, Pvt1, Slc39a14, Map3k11, Brd7, Slc25a28, Tsc1 |
| --- | --- |
