# Supplementary material for: Evidence of pyroptosis and ferroptosis extensively involved in autoimmune diseases at the single-cell transcriptome level
Source: J Transl Med. 2022 Aug 12;20:363. doi: 10.1186/s12967-022-03566-6 (PMC9373312; doi:10.1186/s12967-022-03566-6)
Supplement: Supplementary file 11 — Additional file 11: Table S4. The mouse geneset of ferrotosis suppressor. [file 12967_2022_3566_MOESM11_ESM.docx]

Additional file Table S4. The mouse geneset of ferrotosis suppressor

| Ferrotosis suppressor genes | Gpx4, Rb1, Hspb1, Hsf1, Slc7a11, Gclc, Nfe2l2, Sqstm1, Nqo1, Hmox1, Fth1, Slc3a2, Mt1g, Fancd2, Ftmt, Hspa5, Tp53, Hells, Mtor, Mir137, Slc40a1, Cbs, Otub1, Scd, Sesn2, Nf2, Arntl, Hif1a, Jun, Tmbim4, Plin2, Mir212, Aifm2, Zfp36, Chmp5, Chmp6, Cav1, Pir, Hcar1, Slc16a1, Fzd7, Pla2g6, Park7, Stat3, Acot1, Aldh3a2, Fndc5, Cdh1, Mir214, Tf, Glrx5, Rhebp1, Ppp1r13l, Idh2, Nos2, Rela, Vdr, Fxn |
| --- | --- |
